# Predicted climate shifts within terrestrial protected areas worldwide

**DOI:** 10.1038/s41467-019-12603-w

**Published:** 2019-10-21

**Authors:** Samuel Hoffmann, Severin D. H. Irl, Carl Beierkuhnlein

**Affiliations:** 1 Department of Biogeography, University of Bayreuth, Universitaetsstr. 30, 95447 Bayreuth, Germany; 2 Bayreuth Center of Ecology and Environmental Research, BayCEER, University of Bayreuth, Universitaetsstr. 30, 95447 Bayreuth, Germany; 3 Institute of Physical Geography, Goethe-University, Altenhoeferallee 1, 60438 Frankfurt am Main, Germany; 4 Geographical Institute of the University of Bayreuth, GIB, Universitaetsstr. 30, 95447 Bayreuth, Germany

**Keywords:** Conservation biology, Biogeography, Climate-change ecology

## Abstract

Protected areas (PA) are refugia of biodiversity. However, anthropogenic climate change induces a redistribution of life on Earth that affects the effectiveness of PAs. When species are forced to migrate from protected to unprotected areas to track suitable climate, they often face degraded habitats in human-dominated landscapes and a higher extinction threat. Here, we assess how climate conditions are expected to shift within the world’s terrestrial PAs (*n* = 137,432). PAs in the temperate and northern high-latitude biomes are predicted to obtain especially high area proportions of climate conditions that are novel within the PA network at the local, regional and global scale by the end of this century. These PAs are predominantly small, at low elevation, with low environmental heterogeneity, high human pressure, and low biotic uniqueness. Our results guide adaptation measures towards PAs that are strongly affected by climate change, and of low adaption capacity and high conservation value.

## Abstract (web summary)

Protected areas are important refugia for wildlife, so if climate conditions within them change, wildlife could lose critical suitable habitat. Here the authors calculate the projected gain and loss of climate conditions within terrestrial protected areas worldwide.

## Introduction

Protected areas (PAs) are essential tools to achieve international biodiversity targets^1^. However, anthropogenic climate change will induce a fundamental redistribution of life on earth that affects the effectiveness of PAs^2^ as well as ecosystem functioning and human welfare^3^. Species shift and resize their ranges under climate change, mainly migrating poleward and towards higher elevation as they track suitable habitats^4^. The dynamics of climate change-induced range shifts are in contrast to PAs which are spatially static. As a result, species may lose suitable climatic conditions within PAs and move into unprotected and human-dominated surroundings^5–7^ making extinction rates potentially higher than projected^8^. Currently we lack fine-scale resolution on changing climatic conditions within PAs^9^, particularly at a global extent^6,10–12^.

As the global climate shifts, the climatic conditions found within a given PA may become novel relative to any existing PA (hereafter, “novel climate conditions”). Conversely, climate change may result in the loss of particular combinations of climatic conditions that are represented among the world’s PAs (hereafter, “disappearing climate conditions”). Here, we sought to quantify these gains and losses in climate conditions in the global network of terrestrial PAs. We did so by collating globally available climate (temperature, precipitation)  observations and projections at the 1 km resolution, predicting the temporal change in the spatial distribution of these climate conditions under various emission scenarios, and calculating the percentage of PA land with novel and disappearing climate conditions.

**Fig. 1:**
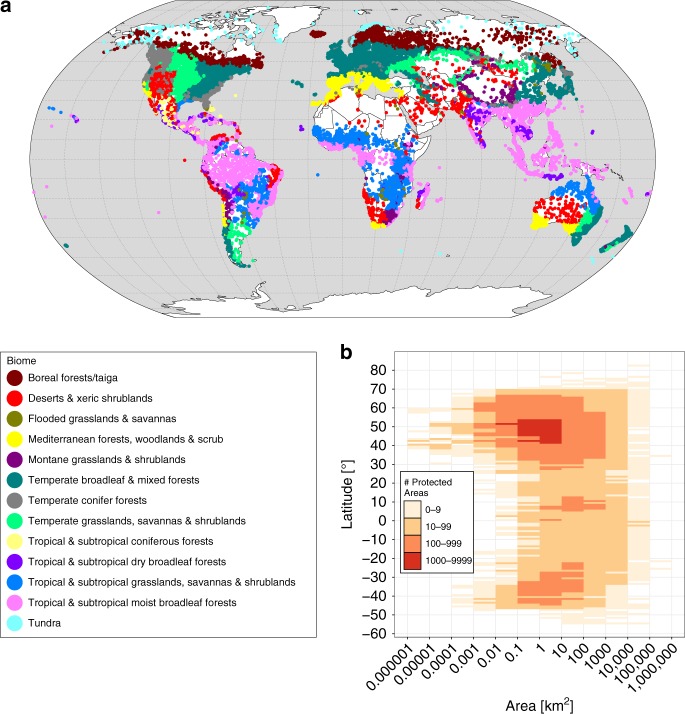
Terrestrial protected areas of the Earth’s biomes. **a** The climate change analyses involve 137,432 terrestrial PAs that cover 20,658,583 km², which is about 14% of the global land area (including Antarctica) and 99.9% of the global PA area. The colored dots represent the centroids of these PAs. The color indicates the biome of the PA. The map was created using open-source software R^52^. **b** The majority of PAs are located between 40° and 50° north. Their area ranges between 0.1 and 10 km²

The percentage of PA land with novel and disappearing climate conditions is expressed by the so-called “novel climate index” and “disappearing climate index”, respectively. We computed the novel and disappaering climate indices for each of 137,432 PAs (Fig. 1). The novel and disappearing climate indices were calculated at three different spatial scales: local, regional, and global. For the local scale, the novel climate index was quantified by the proportion of raster cells of a single PA that hold climate classes in the future scenarios but are currently not present inside the same single PA (i.e. at the local scale). For the regional scale, the novel climate index was quantified by the proportion of raster cells of a single PA that hold climate classes in the future scenarios but are currently not present inside the entire PA network of the respective biome (i.e. at the regional scale). For the global scale, the novel climate index was quantified by the proportion of raster cells of a single PA that hold climate classes in the future scenarios but are currently not present inside the global PA network (i.e. at the global scale). The disappearing climate index was calculated by the proportion of raster cells inside a single PA that hold climate classes currently but are absent in the future scenarios. The novel and disappearing climate indices are estimates of the area proportions of novel or disappearing climate conditions inside individual PAs because the raster cells represent area.

The novel and disappering climate indices were calculated based on the raster cells' current and future climate classes. We, therefore, assigned a current and a future climate class to each raster cell within each PA by applying the algorithm of Carroll et al.^13^. In contrast to other linear, distance-based climate change algorithms (e.g. ref. ^14^), this approach classifies cells in a non-linear fashion with respect to their current and future climate conditions. The climate classifications were based on five independent climate variables that resulted from a principal component analysis (PCA) built on 19 bioclimatic variables. The five-dimensional PCA space (i.e. climate space) was subdivided into climate classes. Each PA raster cell was assigned to a current climate class according to its current climate conditions and to a future climate class according to its future climate conditions.

We calculated the novel and disappering climate indices for the year 2070 accounting for future climate projections of two Representative Concentration Pathways (RCP 4.5 and 8.5) and ten different Global Climate Models (GCM). The RCP scenarios are trajectories for atmospheric greenhouse gas concentrations from the Fifth Assessment Report (AR5) of the International Panel on Climate Change (IPCC). The numbers 4.5 and 8.5 indicate the radiative forcing value in Watt per m^2^. The moderate scenario RCP 4.5 is associated with a mean global temperature increase of 1.8 °C, while the “business-as-usual” scenario RCP 8.5 represents continued high greenhouse gas emission, i.e. a high mean global warming of 3.7 °C by the end of the twenty-first century^15^. We show the mean and standard deviation (Sd) of the PAs’ novel and disappearing climate index throughout the ten GCM for each RCP scenario.

We additionally examined correlations between the local-scale novel and disappearing climate indices and PA characteristics to identify PA attributes that are associated with novel and disappearing climate conditions inside PAs. The PA attributes we examined are area, elevation, topographic heterogeneity (i.e. terrain ruggedness), human pressure (i.e. human footprint), and biotic uniqueness (i.e. irreplaceability). Environmental heterogeneity includes climate diversity and increases with area and topographic heterogeneity. Environmental heterogeneity buffers climate-induced biodiversity loss at the local scale^9,11,16,17^. We expect the degree of climate change inside individual PAs to increase with decreasing environmental heterogeneity (i.e. with decreasing PA size, decreasing elevation, and decreasing terrain ruggedness). This is because completely novel/disappearing climate conditions are less likely under high environmental and climate diversity. Human footprint quantifies anthropogenic land use and habitat loss that can prevent biodiversity conservation under climate change^17^. Irreplaceability is a measure of biotic uniqueness. It quantifies the overlap of PA area with ranges of global Red List species^18^ and, thus, the current conservation value of PAs regarding threatened species worldwide. We assume that the vulnerability of individual PAs to climate change (i.e. the risk of losing irreplaceability under climate change) increases with increasing area of novel/disappearing climate conditions, with decreasing environmental heterogeneity, and with increasing human pressure and irreplaceability. Mean values of the local-scale novel and disappearing climate index were highly correlated (RCP 4.5: Pearson’s coefficient *r* = 0.96, RCP 8.5: *r* = 0.97). We subsequently concentrated on the novel climate index in the main text; see Supplementary Figs. 1–5 for the disappearing climate index.

We find that PAs in the temperate and northern high-latitude biomes are predicted to obtain particularly high area proportions of climate conditions that are novel within the PA network at the local, regional, and global scale. These PAs are predominantly small, at low elevation, with low environmental heterogeneity, high human pressure, and low biotic uniqueness. These results guide adaptation measures towards PAs that are not only strongly affected by climate change, but also of low adaption capacity and high conservation value.

## Results

### Novel climate conditions within PAs

**Fig. 2:**
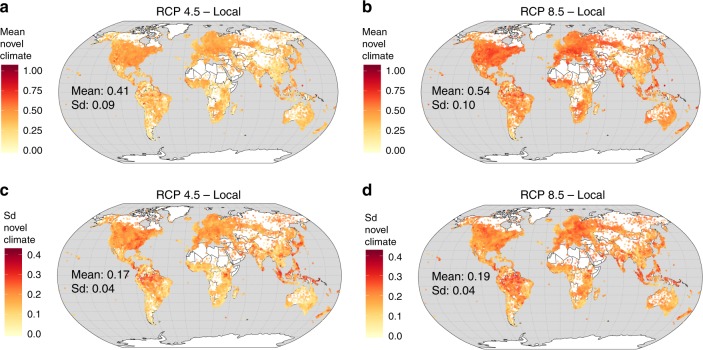
Local-scale novel climate index of terrestrial protected areas worldwide. The local-scale novel climate index shows the proportion of raster cells inside a PA that hold climate classes in the future, which are currently not present in the PA. The mean (**a**, **b**) and standard deviation (**c**, **d**) of the local-scale novel climate index comprise future climate data from ten GCMs under RCP 4.5 and 8.5. Sd represents the variation of the local-scale novel climate index resulting from ten GCMs. **a** Mean of the local-scale novel climate index under RCP 4.5. **b** Mean of the local-scale novel climate index under RCP 8.5. **c** Sd of the local-scale novel climate index under RCP 4.5. **d** Sd of the local-scale novel climate index under RCP 8.5. For each metric in **a**–**d**, the mean and standard deviation across all 137,432 PA values are also given inside the global maps. Data on climate change metrics and other characteristics per PA are given as Supplementary Data 1. The maps were created using open-source software R^52^

**Fig. 3:**
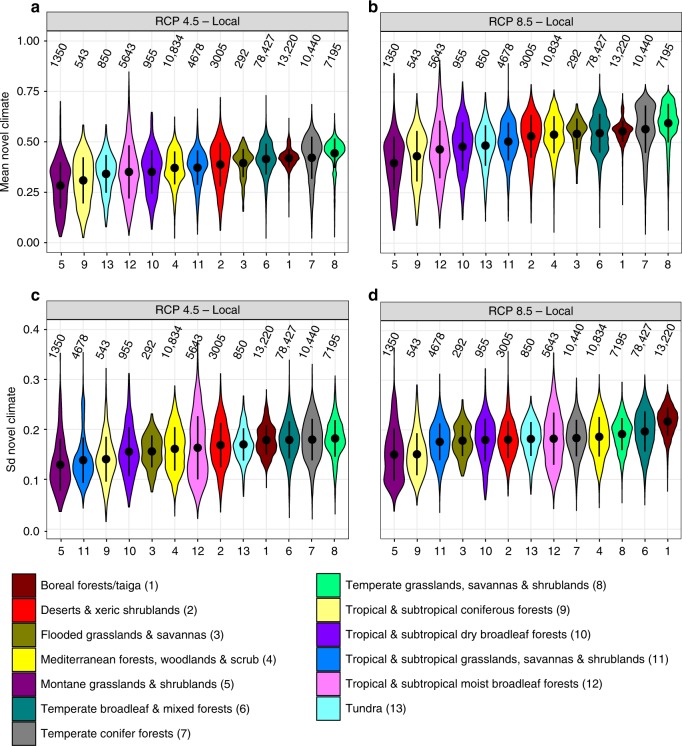
Local-scale novel climate index of terrestrial protected areas worldwide, summarized by biomes. The mean of the local-scale novel climate index under **a** RCP 4.5 and **b** RCP 8.5. The standard deviation (sd) of the local-scale novel climate index under **c** RCP 4.5 and **d** RCP 8.5. Sd represents the variation of the local-scale novel climate index resulting from ten GCMs. Violins per biome are ordered by increasing mean. Black dots and attached lines within violins represent the mean ± standard deviation. Black numbers above violins indicate the number of PAs within the respective biome. Data on climate change metrics and other characteristics per PA are given as Supplementary Data 1. Source data are provided as a Source Data file

PAs could experience on a global average 41% (±9% sd) of local-scale novel climate conditions until 2070, following RCP 4.5, and 54% (±10% sd) according to RCP 8.5 (Fig. 2a, b). The mean values of the local-scale novel climate index are also moderately correlated with the standard deviations of the local-scale novel climate index (Fig. 2c, d; RCP 4.5: *r* = 0.56, *p* < 0.001 using a modified *t*-test accounting for spatial autocorrelation;^19^ RCP 8.5: *r* = 0.43, *p* < 0.001). Under both scenarios, “montane grasslands and shrublands” and “tropical and subtropical coniferous forests” are biomes that include PAs with, on average, the lowest predicted proportions of novel climate conditions at the local scale (Fig. 3a, b). In contrast, the biomes “temperate conifer forests” and “temperate grasslands, savannas and shrublands” contain PAs with the highest predicted proportions of novel climate conditions at the local scale. The standard deviation shows a very similar order at both extremes (Fig. 3c, d).

**Fig. 4:**
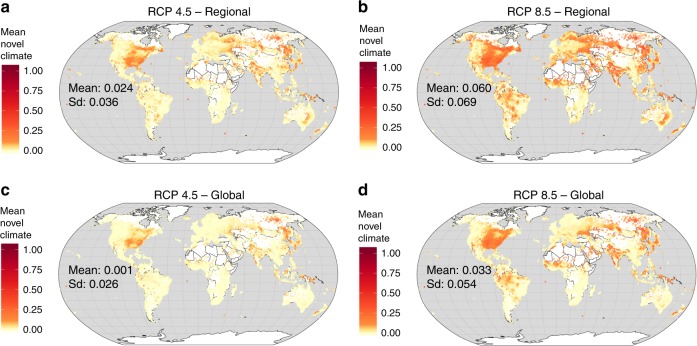
Regional- and global-scale novel climate index of terrestrial protected areas worldwide. The regional-scale novel climate index shows the proportion of raster cells inside a PA that hold climate classes in the future which are currently not present in the entire PA network of the respective biome. The global-scale novel climate index indicates the proportion of raster cells inside a PA that hold climate classes in the future which are currently not present in the global PA network. The mean of the novel climate indices comprise future climate data from ten GCMs under RCP 4.5 and 8.5. **a** Mean of the regional, biome-specific novel climate index under RCP 4.5. **b** Mean of the regional, biome-specific novel climate index under RCP 8.5. **c** Mean of the global-scale novel climate index under RCP 4.5. **d** Mean of the global-scale novel climate index under RCP 8.5. For each metric in **a**–**d** the mean across all 137,432 PA values are also given inside the global maps. Data on climate change metrics and other characteristics per PA are given as Supplementary Data 1. The maps were created using open-source software R^52^

**Fig. 5:**
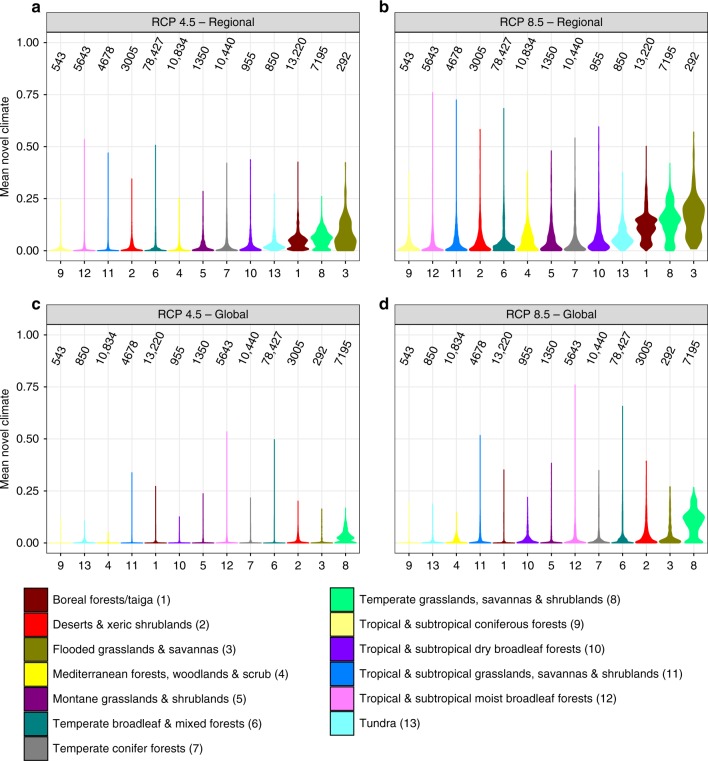
Regional and global-scale novel climate index of terrestrial protected areas worldwide, summarized by biomes. The mean of the regional-scale novel climate index across ten GCMs under **a** RCP 4.5 and **b** RCP 8.5. The mean of the global-scale novel climate index across ten GCMs under **c** RCP 4.5 and **d** RCP 8.5. Violins per biome are ordered by increasing mean. Black numbers above violins indicate the number of PAs within the respective biome. Data on climate change metrics and other characteristics per PA are given as Supplementary Data 1. Source data are provided as a Source Data file

The values of the regional and global-scale novel climate indices demonstrate similar geographical patterns (Figs. 4, 5). The regional-scale index reveals higher values than the global-scale index. The biomes “temperate grasslands, savannas, and shrublands” and “flooded grasslands and savannas” contain PAs with, on average, the highest predicted proportions of novel climate conditions at the regional scale, while “tropical and subtropical moist broadleaf forests” and “tropical and subtropical coniferous forests” contain PAs with the lowest predicted proportions of novel climate conditions at the regional scale (Fig. 5a, b). The biomes “flooded grasslands and savannas” and “temperate grasslands, savannas, and shrublands” include PAs with, on average, the highest predicted proportions of novel climate conditions at the global scale, whereas “tundra” and “tropical and subtropical coniferous forests” include PAs with the lowest predicted proportions at the global scale (Fig. 5c, d). Note, however, that the novel (and disappearing) climate index may over- or underestimate ecological change associated with climate change in some biomes due to the different number of ecoregions within biomes (Supplementary Fig. 8). The climate change metrics may overestimate the ecological change within PAs in “montane grasslands and shrublands”, “temperate broadleaf and mixed forests”, “temperate conifer forests”, “tropical and subtropical coniferous forests” and “tropical and subtropical grasslands, savannas, and shrublands”. The indices may underestimate the ecological change in “deserts and xeric shrublands” and “Mediterranean forests, woodlands, and scrub”. The number and size of PAs (Fig. 1) differ substantially between biomes.

### Relationships between novel climate conditions and PA characteristics

**Fig. 6:**
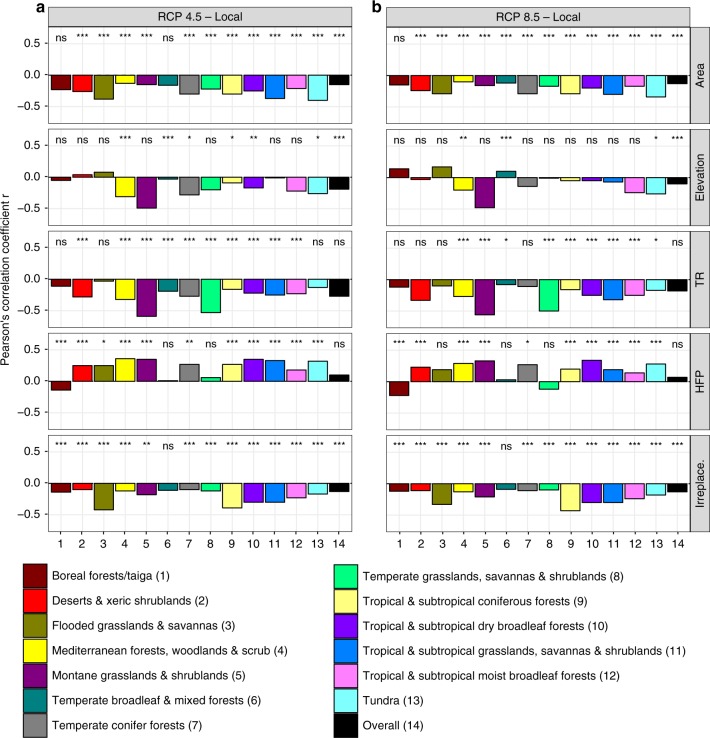
Correlation between the mean values of the local-scale novel climate index and protected area characteristics, separated by biome and RCP scenario. **a** RCP 4.5 and **b** RCP 8.5. Bars show Pearson’s correlation coefficients *r*. Asterisks represent the significance level considering a modified *t*-test accounting for spatial autocorrelation^19^ (*p ≤ 0.05, **p ≤ 0.01, ***p ≤ 0.001), while “ns” implies non-significant (*p* > 0.05) correlation. Note that “TR” stands for terrain ruggedness, “HFP” for human footprint index and “Irreplace.” for irreplaceability. Data on climate change metrics and other characteristics per PA are given as Supplementary Data 1. Source data are provided as a Source Data file

We found negative significant (*p* < 0.05 using a modified *t*-test accounting for spatial autocorrelation^19^) correlations when pooling PAs worldwide (Fig. 6, “overall”): between area (RCP 4.5: *r* = −0.15; RCP 8.5: *r* = −0.13), between elevation (RCP 4.5: *r* = −0.19; RCP 8.5: *r* = −0.1), and between irreplaceability and the local-scale novel climate index (RCP 4.5: *r* = −0.13, RCP 8.5: *r* = −0.13). Even though the global correlations between the local-scale novel climate index and topographic heterogeneity as well as the human footprint show equally high *r*-values for both scenarios, the modified *t*-test revealed no significance due to spatial autocorrelation. Inside individual biomes, the local-scale novel climate index mainly negatively correlates with topographic heterogeneity and positively correlates with the human footprint index.

## Discussion

We found that PAs of temperate and northern high-latitude biomes are predicted to obtain large area proportions of novel climate conditions at the local, regional, and global scale. Large area proportions of novel climate conditions at the regional and global scale could also appear in PAs of flooded grasslands and savannas. PAs that are potentially affected by high proportions of locally novel climate conditions tend to contain low topographic heterogeneity and a large human footprint, suggesting increased vulnerability. However, irreplaceability tends to decrease with an increase in the area proportion of locally novel climate conditions. Hence, PAs that are very important for the conservation of Red List species seem to be less affected by local-scale novel climate conditions.

Novel and disappearing climate conditions indicate novel and disappearing habitat conditions. When PAs gain novel habitats, potentially invasive species might migrate into PAs^20^. When PAs lose habitats, species are likely to migrate out of PAs into unprotected surroundings^5–7^. In both cases, the communities inside PAs are modified with unknown consequences for ecosystem functioning. Since ecosystem functioning depends on biodiversity^21^, the integrity of ecosystems inside PAs is at risk when species diversity decreases through invading and migrating species. Consequently, novel and disappearing climate metrics are basic indicators of such risks.

Our findings can be compared to Loarie et al.^22^, who demonstrated that large PAs in the desert biome will widely retain their current temperature conditions, while small PAs in the Mediterranean biome and in temperate coniferous forests will largely lose their current temperature conditions. Several studies agree that the magnitude of anthropogenic climate change, i.e. the degree of dissimilarity between current and future climate, is predicted to be highest in the tropics, subtropics, and a few northern high-latitude regions^14,22–30^. The (sub-) tropical biomes and northern high-latitude regions could primarily obtain novel, non-analog climates (i.e. future climates without modern analog)^14,24,25,28–30^. The velocity of climate change may be lowest in mountainous regions and highest in continental plains^22,25,29^. Li et al.^26^ illustrated that climate change vulnerability is expected to be highest in plains such as deserts and xeric shrublands, whereas intact boreal and tropical forests, as well as polar regions can be capable of mitigating future climate impacts. These authors revealed that low environmental heterogeneity and small temperature gradients imply high biotic attrition in continental basins under climate change. In addition, areas of high northern latitudes are predicted to become climate-vulnerable in the future. However, a direct comparison of the approach taken by Li et al. with our study is not feasible due to the methodological differences.

We do not indicate that mountain ecosystems inside PAs are less prone to climate change, even though PA land with locally novel climate conditions is predicted to decrease with the increasing elevation of PAs. Climate change may even have a disproportional impact on mountain biomes as exemplified by current melting glaciers and permafrost or increasing mass movements^31^. However, at the landscape scale of mountain PAs, the heterogeneity of site conditions may maintain high biodiversity under climate change, in contrast to PAs of lowland biomes^11^. Mountain PAs play an important role for future biodiversity conservation. They are characterized by large areas, high topographic and, thus, climatic diversity, low human pressure, and high irreplaceability, qualifying them as places for future climate refugia.

We found the numbers and sizes of PAs to explain the area proportions of novel and disappearing climate conditions at the local scale inside PAs. This applies strongly to PAs in the temperate zone that include well-developed industrial nations with high population densities limiting the options to set aside large areas for nature conservation. Temperate biomes exhibit a large number of small PAs at the lower elevation not covering much environmental heterogeneity that could compensate climate change impact. Particularly in the temperate regions of Europe the legacy of land use, high population density, and highly fragmented landscapes are reflected in the establishment of many comparatively small PAs^6^.

The outcomes of this study suggest several implications for conservation action. The negative relationships between the climate change indices and PA attributes such as size, elevation, and topographic heterogeneity emphasize the importance of expanding and establishing large-scale PAs. Such PAs cover high climatic and environmental diversity that can buffer climate change impacts on biodiversity. The biotic uniqueness of PAs is also a major criterion for the conservation value of PAs^32^. PAs showing a high area proportion of locally novel or disappearing climate conditions seem to be less unique for the global conservation of Red List species to date (i.e. low irreplaceability). This relationship may result from the fact that species richness generally decreases towards the poles, while high-latitude regions warm fastest under anthropogenic climate change. However, the positive relationships between the climate change indices and the human footprint indicate that high proportion of human land use will hinder the adaption and migration of species under climate change. These findings should direct policy towards the restoration and maintenance of habitat quality and connectivity, not only within but also between PAs. The co-occurrences of threatened biota, high human pressure, low climate-buffer capacity, and high magnitudes of climate change suggest a high vulnerability of PAs, based on which conservation strategies need to be developed and prioritized^13,24,26^. While the management of PAs varies across the globe, particularly the management effectiveness of climate-vulnerable PAs should be enhanced. Current conservation actions focusing on the management of single habitats and species neglect the majority of biota; revising current conservation policy and pro-active biodiversity management (e.g. habitat restoration, connection, and species translocations) will be essential adaption strategies in view of the climate change velocity^16,33^. We also suggest establishing climate-proof PA networks to overcome the static applications of spatial conservation; climate-proof PA systems implement spatially and temporally dynamic PAs that track the ecological niche of species under climate change^34^. Relocated niches can also be translated into trans-PA conservation schemes^10^. Importantly, early action will be more efficient and less expensive than no or delayed interventions^35^.

Nevertheless, our approach has limitations. Because climate niches of species can extend beyond PAs, novel climate conditions relative to the climate pool of PAs do not necessarily mean the entire habitat for species inside PAs will be lost. Novel climate conditions may have even positive effects, e.g. when threatened species migrate into PAs^2^. We did not consider unprotected surroundings where species may also migrate and persist. Still, PAs are the main tools for biodiversity conservation^18^. Here, we highlight only novel and disappearing climate conditions, but there are many more dimensions of climate change to which species react^25^. The novel and disappearing climate indices do not account for historic inter-annual climate variability. Past inter-annual climate variability increases with latitude and is associated with large-ranged species, while climate stability characterizes areas with many small-ranged species such as those at low latitudes^36^. Small-ranged species, especially those found in the lowland tropics, are at a higher risk of range attrition under climate change than species at higher latitudes^37^. Ecosystems that have experienced high, historical inter-annual climate variability are expected to be more resilient to climate change^9^. However, the effect of inter-annual climate variability on ecosystems can hardly be generalized across ecosystems^38^. Climate data resolution may also underestimate micro-refugia, i.e. local habitats^39^. The detection of climate change velocity inside PAs can additionally foster climate-proof conservation strategies^9^. Furthermore, climate is not the only factor that determines species’ habitats. Habitat can be degraded by other means such as human land use. To integrate all these aspects in future studies and to meet global conservation goals, financial support must increase by at least one order of magnitude^40^.

This study serves as an information resource for climate-smart conservation policy and management from local to global extent. The results can guide the distribution of conservation funds and prioritization. However, recommending an optimal investment strategy for biodiversity conservation under climate change requires a complex analytical framework including ecological and economic factors^41^. High rates of climatic displacement within PAs in the temperate biomes do not suggest focusing conservation effort only here. PAs in less developed countries harbor more biodiversity and are often less effectively managed due to lack of conservation laws, staff, funds, and political willingness^1^. International conservation strategies need to include the demands of a complex setting considering all aspects of climate change as well as biodiversity and socio-economic factors. Nevertheless, it is time to realize the impact of climate change on PAs when discussing conservation policy^42^. Variation in future trends can be quantified, e.g., through the variation in climate models or the deviation between scenarios, but should not be a hindrance for action. For this purpose, it is important to increase societal and political awareness about the consequences of climate change for biodiversity and human well-being.

## Methods

### Protected area data

The World Database on Protected Area (WDPA)^43^ includes boundary (polygon) data for 201,464 purely terrestrial designated PAs. These PAs cover 20,702,558 km², amounting to around 15% of the Earth’s land surface. We rasterized these PA polygons in the same resolution as the climate data (30 arc seconds, i.e. approx. 900 m at the equator) via cell center coverage. Thus, relatively small PAs and PAs which have an elongated shape may cover only a few or even no raster cells. After rasterization, 137,735 PAs remained, from which another 303 PAs were excluded because the centroids of those 303 PAs were located in the “Mangroves” biome^44^ and are consequently assumed to be coastal PAs. Eventually, we considered 26,038,594 cells that are covered by 137,432 PAs, which still comprise a total area of 20,658,583 km^2^ (i.e. 14% of the global terrestrial surface and 99.9% of all PA area). We refer to these raster cells as “protected cells”.

To identify PA attributes that are particularly associated with climate change inside PAs, we related several PA characteristics to the novel and disappearing climate index. We assigned each PA to its biome by overlaying the PA centroids and the biome polygons provided by Olson et al.^44^. The biome informs us about dominant ecosystem types. The PA area is given by the WDPA. The size of the PA influences the number of resources for species’ adaption and migration under climate change. We extracted the median elevation of each PA from a digital elevation model with a resolution of 30 arc seconds provided by Amatulli et al.^45^. The median elevation indicates the geographical location of PAs in highland or lowland regions. The Terrain Ruggedness Index (TR) is a measure of topographic heterogeneity. This product is based on 90 m elevation data from the Shuttle Radar Topography Mission and has a final resolution of 30 arc seconds^45^. The TR was calculated as the mean of the absolute differences in elevation between a protected cell and its eight adjacent protected cells. Planar area has a TR of 0 m. The TR of mountain areas can be as high as 2000 m in the Himalaya region^45^. We used the median of the TR values inside PAs to represent the topographic heterogeneity of each PA. Topographic heterogeneity implies elevational gradients as well as climatic and habitat heterogeneity. Topographic heterogeneity reflects the adaptive capacity of PAs’ biodiversity to impacts of climate change^16^. The probability of species tracking suitable environmental conditions within the same PA—via adaptation or migration—is higher in areas with more heterogeneous conditions. Environmental heterogeneity buffers climate change effects on ecosystems^9^. The human footprint index 2009 comprises eight indicators of human impact on natural systems that stem from in situ and remotely sensed data^46^: population density, buildings, electric infrastructure, roads, railways, navigable waterways, cropland, and pasture. The human footprint of a PA was calculated by the median human footprint of the raster cell values that fall within the PA. The irreplaceability of PAs is a measure of biotic uniqueness and quantifies the degree of overlap between each PA and the range of species of the IUCN Red List^18^. In total, 21,419 species were considered: 6240 amphibians, 9793 birds, and 5263 mammals. Since irreplaceability was calculated for the WDPA (Version October 2012), we could link the irreplaceability index to our PA data by the WDPA ID.

### Climate data

We used global climate data with a resolution of 30 arc seconds provided by the WorldClim project (Global Climate Data Version 1.4; Hijmans et al.^47^). Current climate data were produced by interpolations of observed data of the time period between 1960 and 1990. Future climate data were downscaled from GCMs to the Coupled Model Intercomparison Project Phase 5 (Intergovernmental Panel on Climate Change, Fifth Assessment Report). We implemented the Representative Concentration Pathway RCP 4.5 and 8.5, and the following global climate models (GCM) for the year 2070, i.e. the average of period 2061–2080: BCC-CSM1–1, CCSM4, CNRM-CM5, GFDL-CM3, HadGEM2-AO, INMCM4, IPSL-CM5A-LR, MIROC5, MPI-ESM-LR, and MRI-CGCM3. We chose pathways RCP 4.5 and 8.5 because they delimit a range of future climate conditions that are likely to occur. We only considered raster cells that hold information about each of the 19 bioclimatic variables provided for current and future climate conditions. We refer to these raster cells as “climate cells” hereafter.

### Climate change analyses

We calculated the novel and disappearing climate indices of each PA for each GCM and RCP. The calculation of the novel and disappearing climate indices is only based on climate cells that are covered by a PA (“protected climate cells”). One reason for this approach is that PAs are expected to be the only remaining and isolated sites for global biodiversity conservation in future^48^. Another reason refers to the enormous computing capacity that would be required when considering the climate pool of the global land surface at a spatial resolution of 1 km. Nevertheless, the climate pool of the global PA network well represents the climate pool of the global land surface because the PAs have worldwide distribution (Fig. 1). However, this approach entails disadvantages that are discussed in the main text.

To identify protected climate cells that considerably change climate conditions between present and future, we adapted the algorithm of Carroll et al.^13^, which is based on Hamman et al.^49^. Accordingly, we applied a Principal Component Analysis (PCA). We constructed the PCA space based on a random sample of 10,000,000 (i.e. 40% of the total amount of protected climate cells). This representative sample is still computationally manageable. The random sampling implies that each climate type is sampled proportionally to its extent. The PCA was built on both current and future climate information because all possible climate conditions now and in the future are supposed to be represented by the PCA^10^. We only considered the first five PCA axes for further analysis to reduce the climate information from 19 bioclimatic variables to five independent variables. As an example, the first five PCA axes partially built on future climate data from BCC-CSM1-1 under RCP 8.5 account for 92% of the variation in the 19 original bioclimatic variables. These first five axes correspond to thermal and hydraulic variables (Supplementary Tables 1 and 2). We then predicted for each protected climate cell the current and future position on the first five PCA axes. Subsequently, each protected climate cell received a current and future position in the five-dimensional climate space. The five-dimensional climate space was then subdivided into climate classes. To create those classes, each of the first five PCA axes was subdivided into equally sized bins. Then, the bins along each axis were grouped according to their spatial intersection in the five-dimensional space. Each group of intersecting bins finally was taken to constitute a climate class. Each climate cell could now be assigned to a current climate class based on the cell’s current position in the PCA space and to a future climate class based on the cell’s future position in the PCA space. Hence, each cell holds a current and future climate class. If current and future cell positions fall within the same class, it is assumed the climate of that cell will barely change. Since the delimitation of classes in the five-dimensional PCA space is crucial for the result, we randomly shifted the bin limits 30 times around the actual bin limits within the range of the bin width, and took the mean of the 30 different outcomes (adapted from Carroll et al.^13^).

This non-linear classification approach needs less computing capacity and time than classic and linear distance methods (e.g. ref. ^14^) because in the non-linear classification method there is no need to calculate pairwise distances between very large numbers of grid cells^49^. Very large numbers of grid cells are given when the spatial resolution is high. However, this non-linear classification approach has several drawbacks. It does not account for distance or dissimilarity between current and future climate conditions in an ordinal way because ordinal distances between climate classes are not applied. The non-linear classification algorithm does also not incorporate historic inter-annual climate variability, which could improve the assessment of future climate distance/dissimilarity^14^.

The number of PCA axes considered and the bin width used for subdividing the PCA axes determines the total number of climate classes. The novel and disappearing climate indices are sensitive to the number of axes and the bin width because the indices are based on the number of climate classes. Carroll et al.^13^ state that applying five axes and a bin width of 2 PCA units are appropriate for the Western hemisphere. Here, we conducted another sensitivity analysis that demonstrated the relationship between the bin width and the resulting number of climate classes given by five PCA axes. We accounted for five PCA axes because they explain 92% of the variation in the original climate data and are still computationally manageable. For the sensitivity analysis, climate data from BCC-CSM1-1 under RCP 8.5 were taken as an example (Supplementary Fig. 7). The more climate classes are defined, the more sensitive are the indices (i.e. the higher are the index values). In our example, a bin width of 2 PCA units yielded 430 climate classes worldwide (Supplementary Fig. 7a, red line); 320 classes are defined by current climate conditions, and 372 by future conditions; present and future conditions shared 262 classes. The threshold of 2 PCA units (Supplementary Fig. 7b, red line) balances underestimation of climate change by very broad climate classes and overestimation of climate change by very narrow classes. Consequently, we agree with Carroll et al.^13^ not only for reasons of comparability, and use five PCA axes and a PCA bin width of 2 PCA units. Additionally, the resulting climate change metrics depend on the number of climate variables put into the PCA and the spatial resolution of climate data. The geographic patterns of climate change estimates, however, are robust against these user choices^49^. Thus, it makes this approach useful for prioritizing conservation management.

To assess the degree of ecological differentiation between climate classes resulting from a bin width of 2 PCA units, we compared the number of climate classes to the number of ecoregions worldwide^44^. Taking again BCC-CSM1-1 under RCP 8.5 as an example, 320 classes were calculated for current climate conditions worldwide. Olson et al.^44^ describe 867 present ecoregions nested within 14 biomes. Because the ecoregion richness is almost triple the number as the number of current climate classes, our climate change metrics underestimate, at the global scale, the ecological change that is associated with changes of the climate class. However, at the biome scale, the relation between the number of ecoregions and current climate classes may be different. We, therefore, related the number of climate classes to the number of ecoregions per biome. This comparison serves as a caveat that our metrics may overestimate climate-induced ecosystem change in some biomes and underestimate in others. Because of the number of ecoregions deviating from the number of current climate classes to more than 25% (Supplementary Fig. 8), the climate change metrics may considerably overestimate the ecological change within PAs in “montane grasslands and shrublands”, “temperate broadleaf and mixed forests”, “temperate conifer forests”, “tropical and subtropical coniferous forests” and “tropical and subtropical grasslands, savannas, and shrublands”, and underestimate in “deserts and xeric shrublands” and “Mediterranean forests, woodlands, and scrub”.

Our cell classification procedure allows for the calculation of a variety of climate change indices. Here we focused on two indices of fundamental importance^9,14,50,51^: the novel climate index and the disappearing climate index. We calculated the novel climate index and the disappearing climate index for each PA at the local, regional, and global scale. We defined the local-scale novel climate index as the proportion of cells within a single PA that hold climate classes in the future projections that do not currently exist within the same single PA (i.e. at the local scale). We defined the regional-scale novel climate index as the proportion of cells within a single PA that hold climate classes in the future projections that do not currently exist within the entire PA network of the respective biome (i.e. at the regional scale). We defined the global-scale novel climate index as the proportion of cells within a single PA that hold climate classes in the future projections that do not currently exist within the global PA network (i.e. at the global scale). The disappearing climate index was calculated by the proportion of cells within a single PA that currently hold climate classes that do not exist in the future. The climate of a protected climate cell can be novel and disappearing at the same time. The indices are based on cell counts and do not hold any unit. Since the raster cells represent an area, they can be perceived as an estimate of a proportional area of the novel or disappearing climate conditions inside individual PAs. The local-scale novel and disappearing climate indices are more sensitive indicators of climate change than the regional and global-scale indices because the local-scale indices were calculated based on a smaller geographical extent including fewer climate classes. The fewer climate classes are found inside a geographical extent, the more likely are novel and disappearing climate classes inside this extent, which will increase the novel and disappearing climate indices. The regional- and global-scale indices of disappearing climate conditions are less sensitive indicators of climate change because they represent the area proportions of climate inside a PA that is in future not only lost from the PA (local scale) but also from the entire PA network of the biome (regional scale) or from the global PA network (global scale). Both indices exhibit several weaknesses for the benefit of computational feasibility: they do not represent climate distance or dissimilarity in an ordinal way. While they indicate the area proportions of novel and disappearing climate conditions, they do not show how dissimilar the future climate will be compared to the current climate. Furthermore, the metrics do not account for historic inter-annual climate variability. Inter-annual climate variability increases with latitude and is associated with large-ranged species, while climate stability characterizes areas with small-ranged species such as those at low latitudes^36^. The effects of inter-annual climate variability on ecosystems cannot be generalized and depend on the current ecosystem state^38^. Small-ranged species, however, are at particular risk of range attrition under global warming^37^. We calculated the mean and standard deviation of the indices over ten GCMs per RCP. The standard deviation and value range between RCP 4.5 and 8.5 are estimates of variation, i.e. uncertainty among climate predictions.

### Statistical analyses

We tested for correlations between climate change indices as well as between climate change indices and PA characteristics by using Pearson’s correlation coefficient *r* and a modified *t*-test accounting for spatial autocorrelation^19^.

### Reporting summary

Further information on research design is available in the Nature Research Reporting Summary linked to this article.

## Supplementary information

Supplementary Information

Peer Review File

Reporting Summary

Description of Additional Supplementary Files

Supplementary Data 1

Source Data

## Data Availability

All data used in this study are open. Data produced in this study are attached as Supplementary Data 1 and stored in the figshare repository (10.6084/m9.figshare.9804350). See Carroll et al.^13^ for R code. The source data underlying Figs. 3, 5, and 6 as well as Supplementary Figs. 2, 4, 5, 6, 7, and 8 are attached as a Source Data file.

## Code availability

The algorithm we used to calculate climate change is adapted from Carroll et al^13^.
