# Supplementary material for: Predicted climate shifts within terrestrial protected areas worldwide
Source: Nat Commun. 2019 Oct 21;10:4787. doi: 10.1038/s41467-019-12603-w (PMC6803628; doi:10.1038/s41467-019-12603-w)
Supplement: Supplementary file 1 — Supplementary Information [file 41467_2019_12603_MOESM1_ESM.pdf]

Supplementary Information for

Predicted climate shifts within terrestrial protected areas worldwide

Samuel Hoffmann[1], Severin D.H. Irl[1,2,3], Carl Beierkuhnlein[1,2,4]

[1] Department of Biogeography, University of Bayreuth, Universitaetsstr. 30, 95447 Bayreuth, Germany
[2] Bayreuth Center of Ecology and Environmental Research, BayCEER, University of Bayreuth, Universitaetsstr. 30, 95447 Bayreuth, Germany
[3] Institute of Physical Geography, Goethe-University, Altenhoeferallee 1, 60438 Frankfurt am Main, Germany
[4] Geographical Institute of the University of Bayreuth, GIB, Universitaetsstr. 30, 95447 Bayreuth, Germany

Corresponding Author: Samuel Hoffmann, Department of Biogeography, University of Bayreuth, Universitaetsstr. 30, 95447 Bayreuth, Germany, E-mail: samuel.hoffmann@uni-bayreuth.de, Phone: +49921-552299

This file includes:
Supplementary Figure 1 to 8
Supplementary Tables 1 to 2
Supplementary References

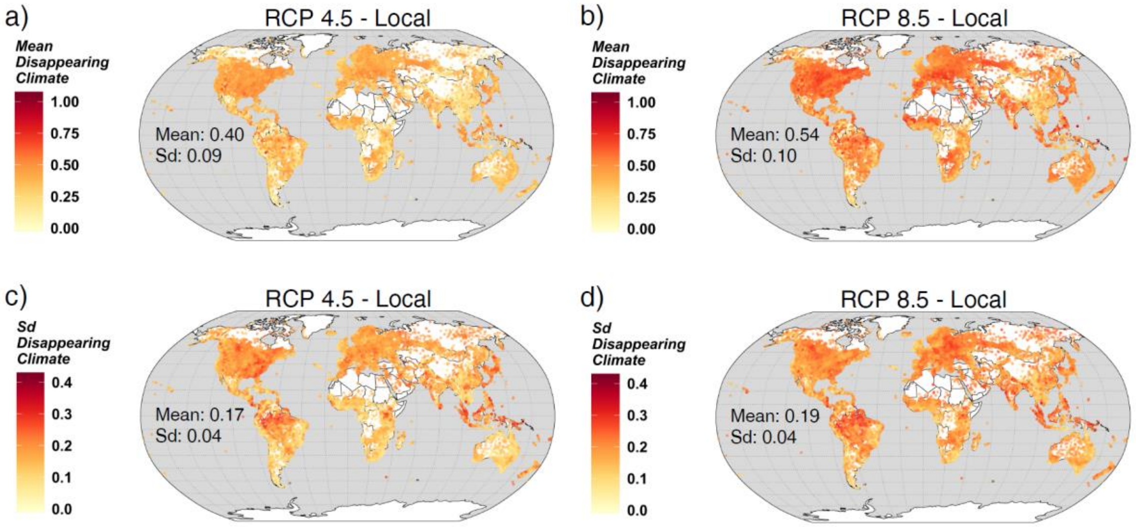

Supplementary Figure 1. Local-scale disappearing climate index of terrestrial protected areas worldwide. The local-scale disappearing climate index shows the proportion of raster cells inside a PA that currently hold climate classes, which will disappear from the PA in the future. The mean and standard deviation (sd) of the local-scale disappearing climate index comprise future climate data from ten GCMs under RCP 4.5 and 8.5. Sd represents the variation of the local-scale disappearing climate index resulting from ten GCMs. a) Mean of the local-scale disappearing climate index under RCP 4.5. b) Mean of the local-scale disappearing climate index under RCP 8.5. c) Sd of the local-scale disappearing climate index under RCP 4.5. d) Sd of the local-scale disappearing climate index under RCP 8.5. For each metric in a) to d), the mean and standard deviation across all 137,432 PA values are also given inside the global maps. Data on climate change indices and other characteristics per PA are given as Supplementary Data 1. The maps were created using open-source software R [1].

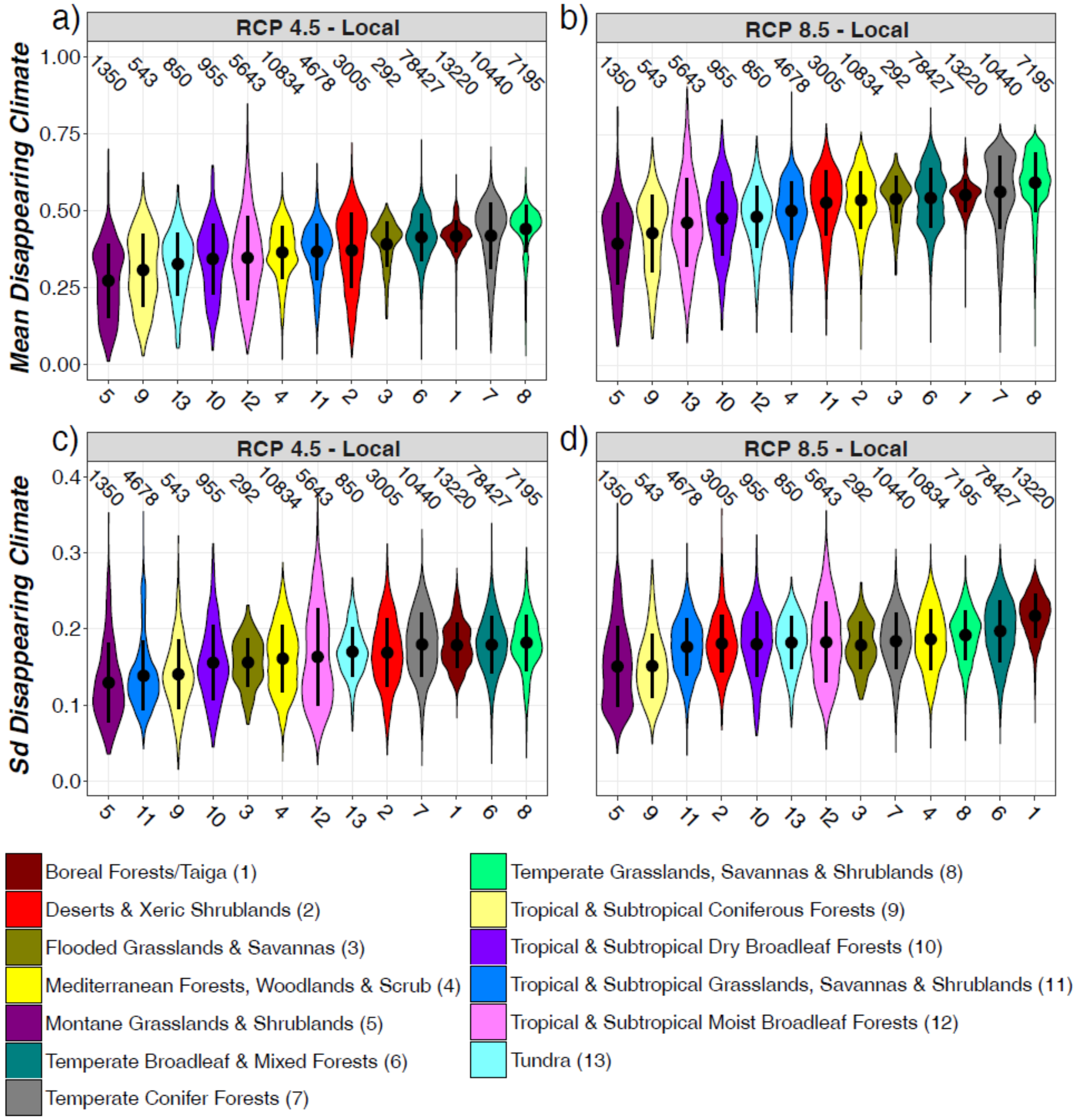

Supplementary Figure 2. Local-scale disappearing climate index of terrestrial protected areas worldwide, summarized by biomes. The mean of the local-scale disappearing climate index across ten GCMs under a) RCP 4.5 and b) RCP 8.5. The standard deviation (sd) of the local-scale disappearing climate index across ten GCMs under c) RCP 4.5 and d) RCP 8.5. Violins per biome are ordered by increasing mean. Black dots and attached lines within violins represent the mean ± standard deviation. Black numbers above violins indicate the number of PAs within the respective biome. Source data are provided as a Source Data file.

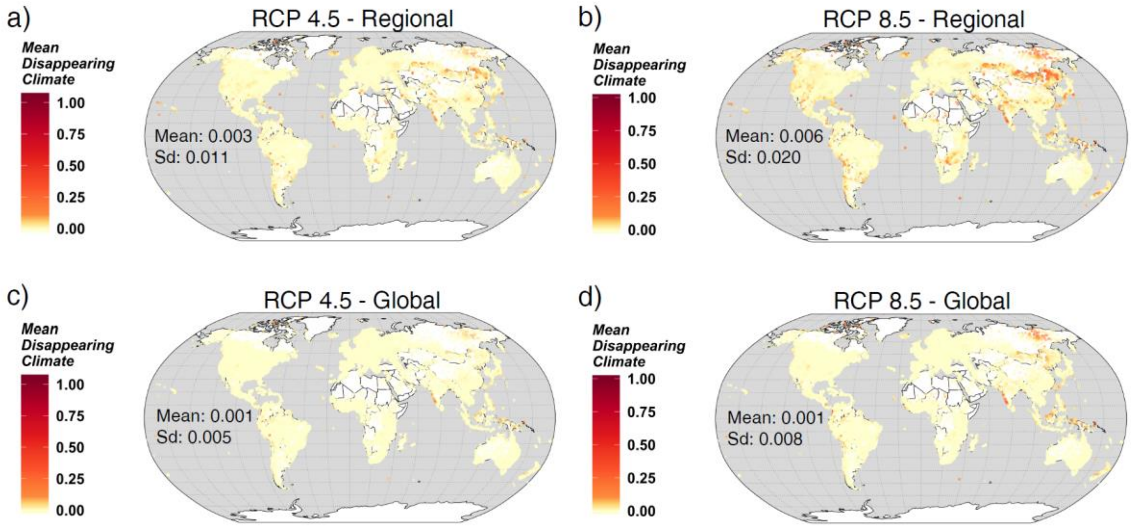

Supplementary Figure. 3. Regional and global-scale disappearing climate index of terrestrial protected areas worldwide. The regional-scale disappearing climate index shows the proportion of raster cells inside a PA that currently hold climate classes, which will disappear from the entire PA network of the respective biome in the future. The global-scale disappearing climate index indicates the proportion of raster cells inside a PA that currently hold climate classes, which will disappear from the global PA network in the future. The mean of the disappearing climate index comprise future climate data from ten GCMs under RCP 4.5 and 8.5. a) Mean of the regional-scale disappearing climate index under RCP 4.5. b) Mean of the regional-scale disappearing climate index under RCP 8.5. c) Mean of the global-scale disappearing climate index under RCP 4.5. d) Mean of the global-scale disappearing climate index under RCP 8.5. For each metric in a) to d) the mean across all 137,432 PA values are also given inside the global maps. Data on climate change indices and other characteristics per PA are given as Supplementary Data 1. The maps were created using open-source software R [1].

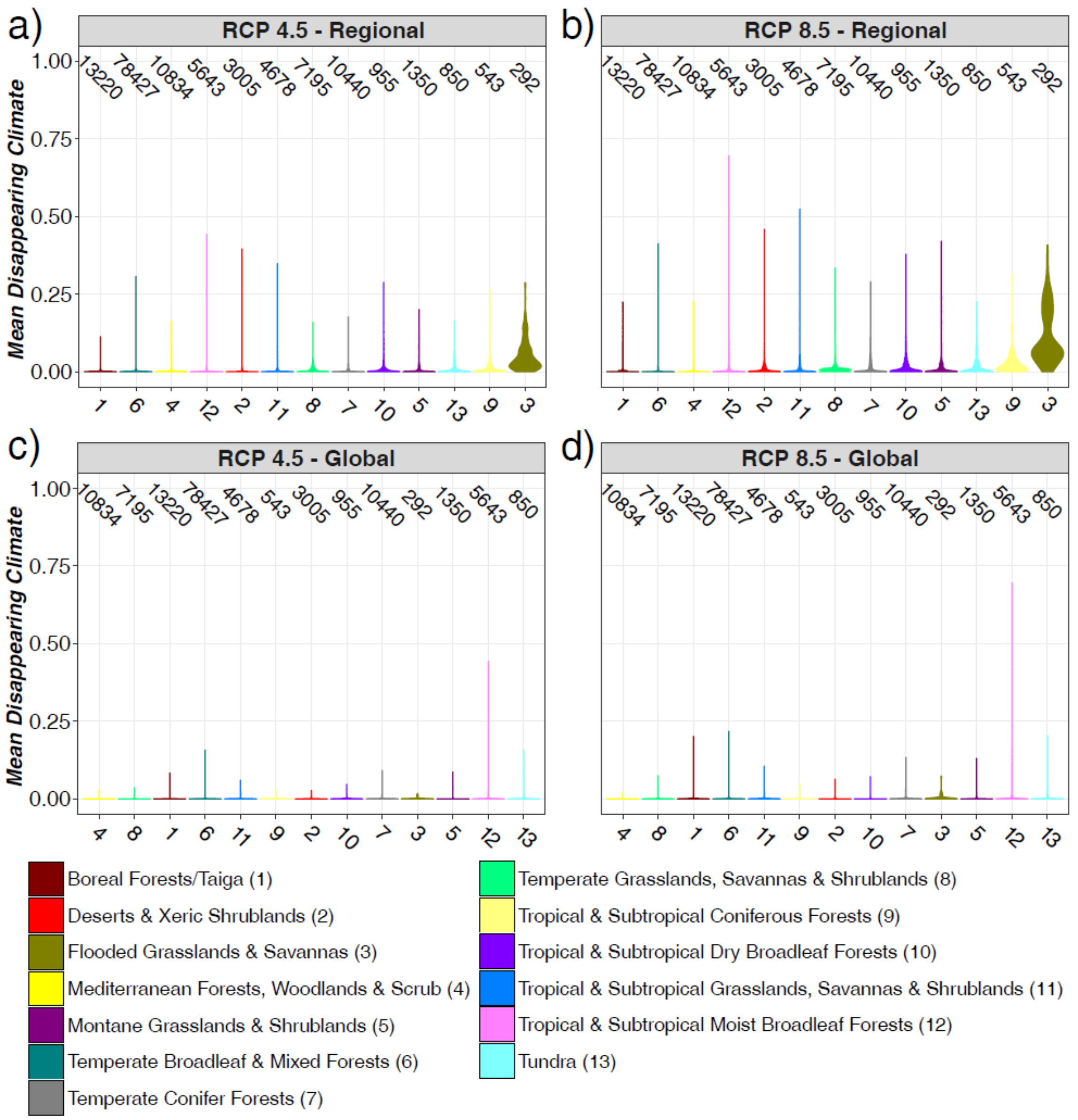

Supplementary Figure 4. Regional and global-scale disappearing climate index of terrestrial protected areas worldwide, summarized by biomes. The mean of the regional-scale disappearing climate index across ten GCMs under a) RCP 4.5 and b) RCP 8.5. The mean of the global disappearing climate index across ten GCMs under c) RCP 4.5 and d) RCP 8.5. Violins per biome are ordered by increasing mean. Black numbers above violins indicate the number of PAs within the respective biome. Source data are provided as a Source Data file.

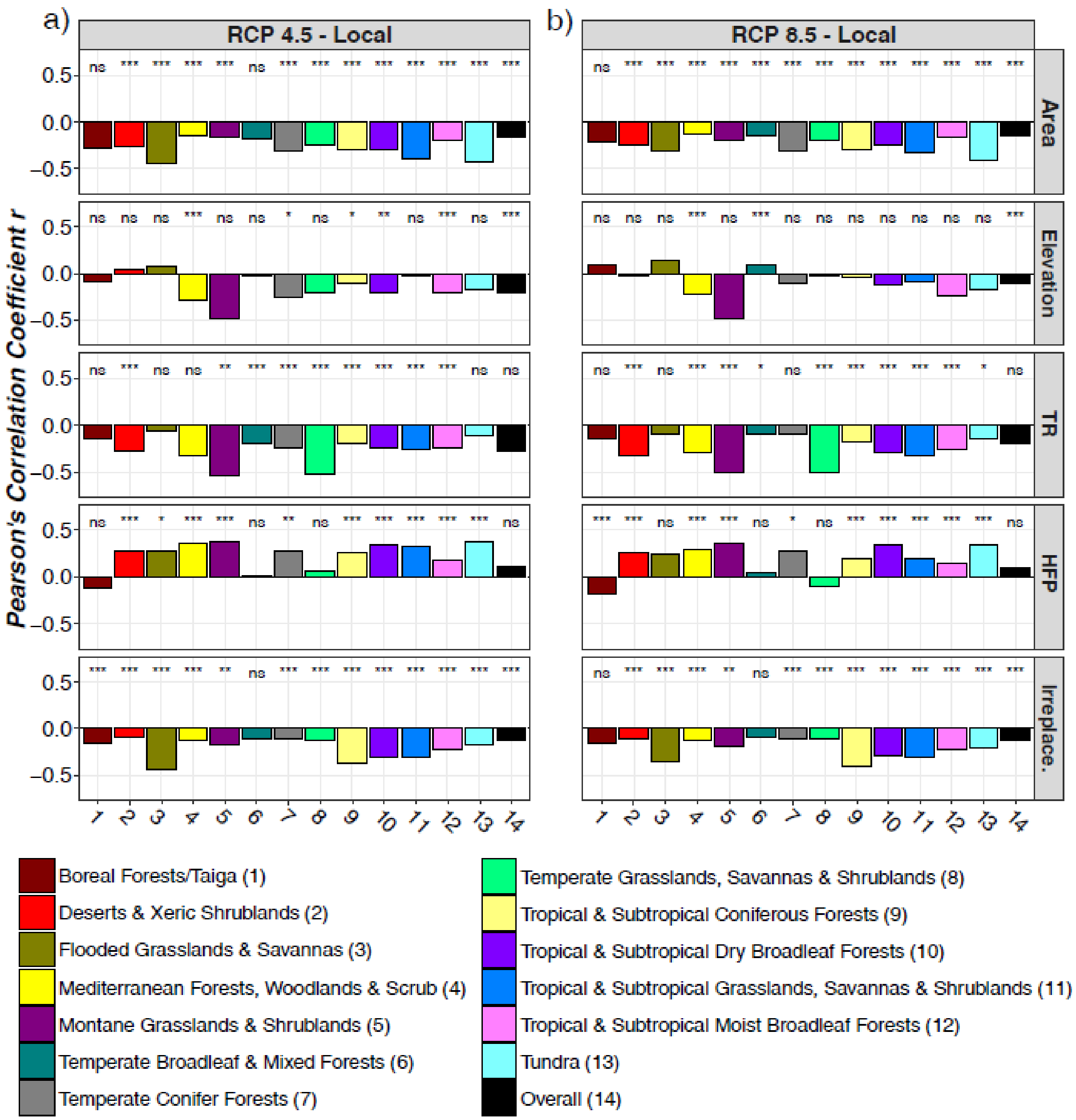

Supplementary Figure. 5. Correlation between the mean values of the local-scale disappearing climate index and protected area characteristics, separated by biomes and RCP scenarios. a) RCP 4.5 and b) RCP 8.5. The proportion of local-scale disappearing climate inside PAs increases with decreasing area, elevation, topographic heterogeneity (terrain ruggedness [TR]) and irreplaceability (Irreplace.), and with increasing human footprint (HFP), globally and to a varying degree among biomes. Bars show Pearson's correlation coefficients *r*. Asterisks represent the significance level considering a modified t-test accounting for spatial autocorrelation [2] (*: $p \leq 0.05$, **: $p \leq 0.01$, ***: $p \leq 0.001$), while 'ns' implies non-significant ($p > 0.05$) correlation. Source data are provided as a Source Data file.

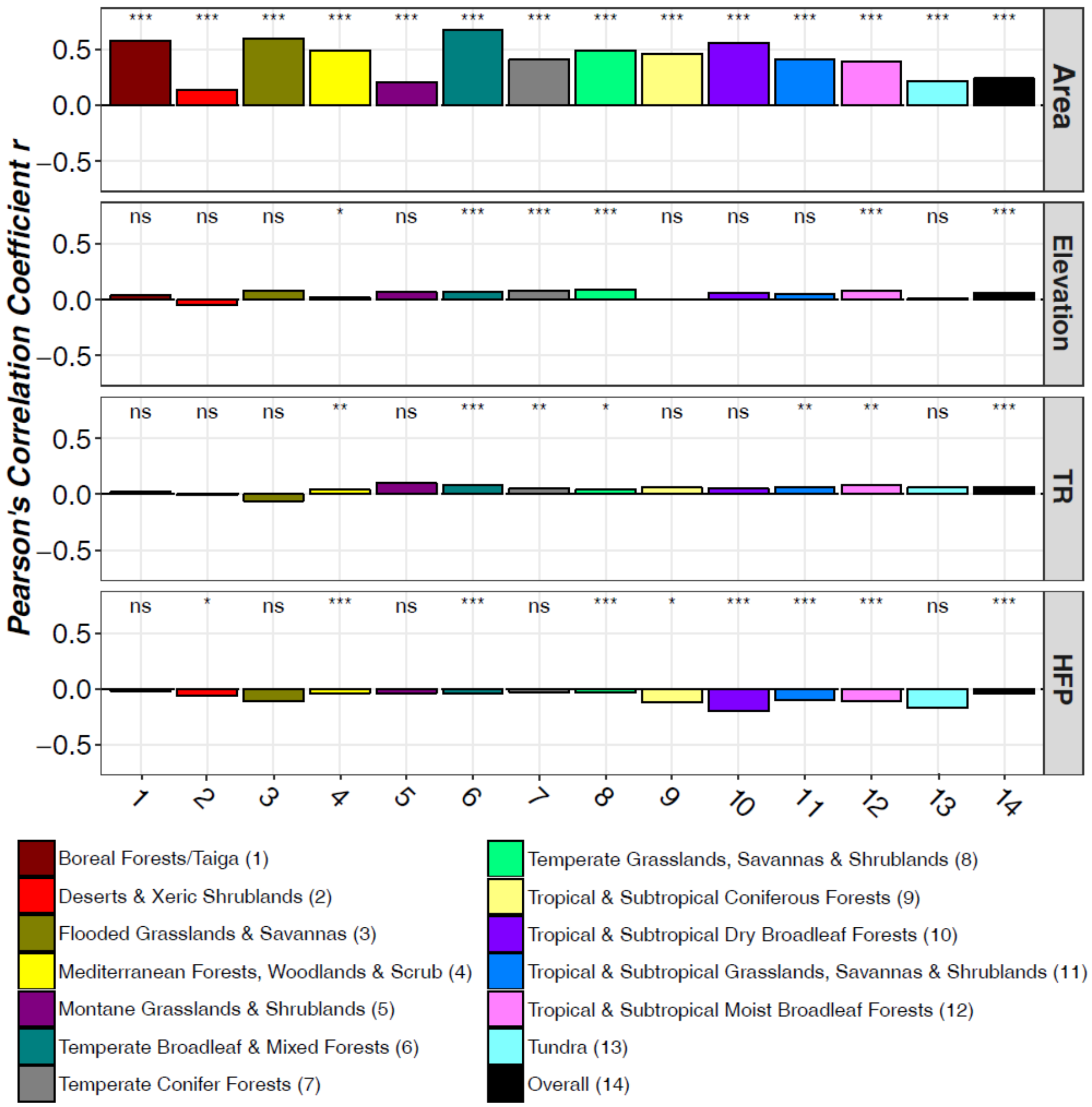

Supplementary Figure 6. Correlation between the protected areas' irreplaceability and other characteristics, separated by biome. Irreplaceability increases with area, elevation and topographic heterogeneity (terrain ruggedness [TR]), and decreases with human footprint (HFP) size, globally and to a varying degree among biomes. Bars show Pearson's correlation coefficients *r*. Asterisks represent the significance level considering a modified t-test accounting for spatial autocorrelation [2] (*: $p \leq 0.05$, **: $p \leq 0.01$, ***: $p \leq 0.001$), while 'ns' implies non-significant ($p > 0.05$) correlation. Source data are provided as a Source Data file.

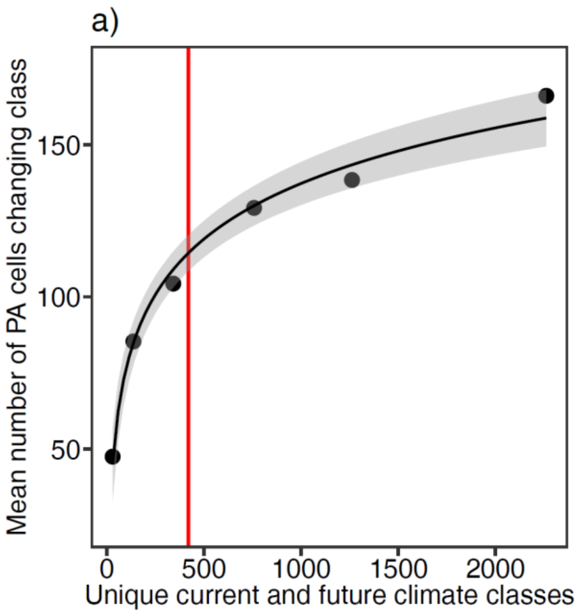

Supplementary Figure 7. Sensitivity of changing cell classes to climate class width. a) Relationship between the defined number of current and future climate classes and the mean number of cells that change climate class per protected area worldwide. b) Relationship between the defined class bin width in PCA space and the mean number of cells that change climate class per protected area worldwide. The red line indicates a PCA climate bin width of 2 PCA units, which results in 430 current and future climate classes. The black lines are logarithmic functions fitted to the data (the grey band equals the 95% confidence interval).

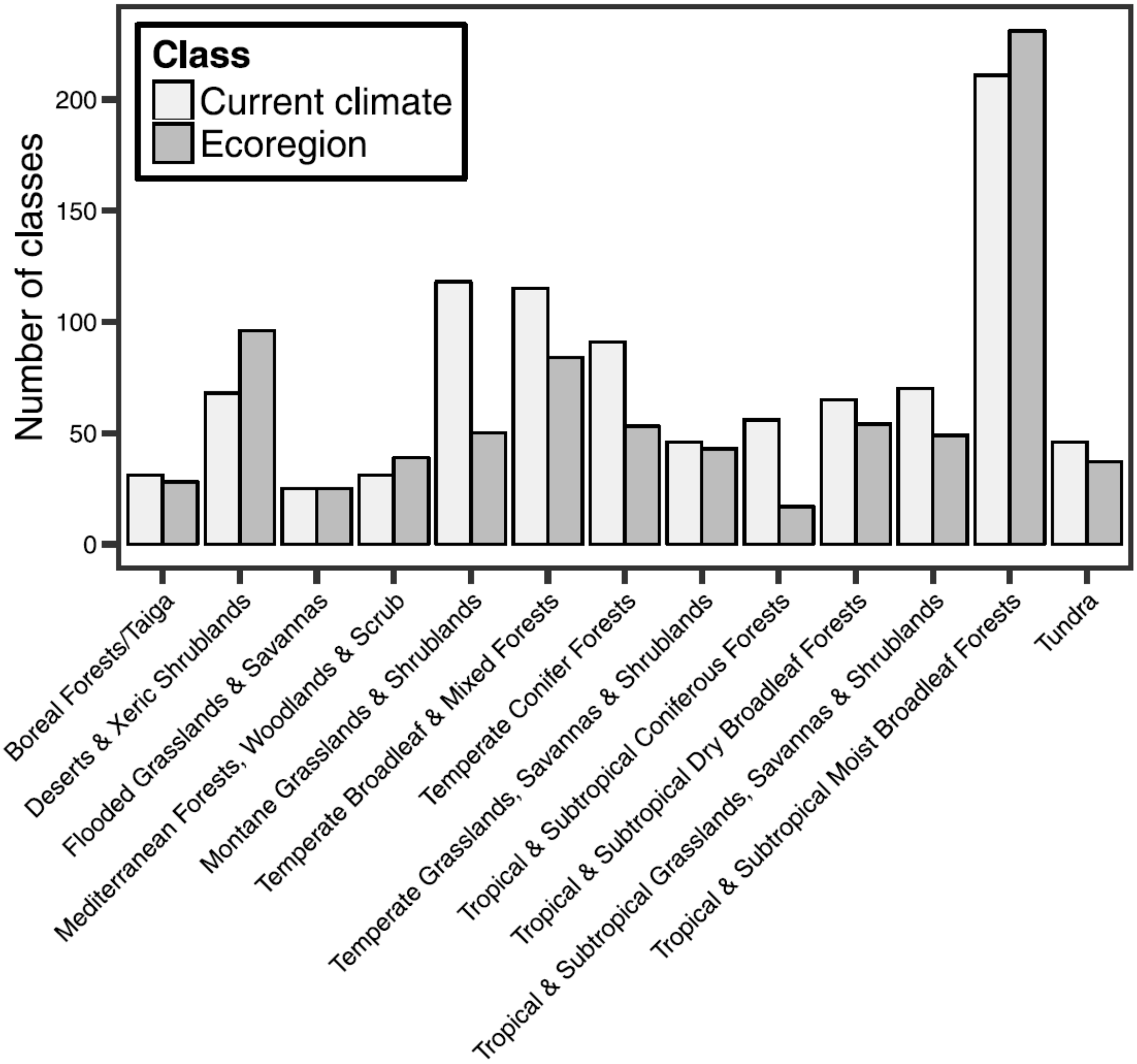

Supplementary Figure 8. Comparison between the number of current climate classes applied in this study and the number of ecoregions described by Olson et al. [3] inside terrestrial biomes. The positive and negative differences between both numbers indicate respectively over- and underestimation of ecological change within biomes that are associated with climate class changes over time and resulting climate change metrics.

Supplementary Table 1. Exemplary PCA outcomes the climate change indices are resulting from. The standard deviation and variance of the first eleven PCA axes are shown. As an example, this PCA is based on future climate data from the Global Climate Model BCC-CSM1-1 for RCP 8.5 and the year 2070, i.e. the average of the time period 2061-2080.

	PC1	PC2	PC3	PC4	PC5	PC6	PC7	PC8	PC9	PC10	PC11
**Standard deviation**	4.539	2.970	1.593	1.385	1.091	1.031	0.812	0.649	0.429	0.351	0.327
**Proportion of variance**	0.542	0.232	0.067	0.050	0.031	0.028	0.017	0.011	0.005	0.003	0.003
**Cumulative proportion**	0.542	0.774	0.841	0.892	0.923	0.951	0.968	0.979	0.984	0.987	0.990

Supplementary Table 2. Exemplary PCA outcomes the climate change indices are resulting from. The PCA loadings of the first five axes are shown, which are used for the calculation of climate change indices. As an example, this PCA is based on future climate data from the Global Climate Model BCC-CSM1-1 for RCP 8.5 and the year 2070, i.e. the average of the time period 2061-2080.

Time	Bioclimatic variable	PC1	PC2	PC3	PC4	PC5
Current	Annual Mean Temperature	-0.204	-0.119	-0.066	-0.012	0.037
Current	Mean Diurnal Range	-0.022	-0.255	0.019	-0.144	-0.501
Current	Isothermality	-0.201	-0.029	0.017	0.120	-0.176
Current	Temperature Seasonality	0.201	0.027	0.026	-0.267	0.095
Current	Max Temperature of Warmest Month	-0.157	-0.193	-0.098	-0.244	0.097
Current	Min Temperature of Coldest Month	-0.211	-0.067	-0.073	0.106	0.038
Current	Temperature Annual Range	0.196	-0.028	0.040	-0.302	0.008
Current	Mean Temperature of Wettest Quarter	-0.162	-0.143	0.027	-0.250	0.134
Current	Mean Temperature of Driest Quarter	-0.199	-0.096	-0.117	0.085	0.007
Current	Mean Temperature of Warmest Quarter	-0.173	-0.165	-0.091	-0.211	0.144
Current	Mean Temperature of Coldest Quarter	-0.209	-0.089	-0.056	0.091	-0.001
Current	Annual Precipitation	-0.172	0.191	0.098	-0.067	0.004
Current	Precipitation of Wettest Month	-0.174	0.122	0.270	-0.046	0.088
Current	Precipitation of Driest Month	-0.099	0.252	-0.200	-0.126	-0.222
Current	Precipitation Seasonality	-0.006	-0.212	0.388	0.050	-0.202
Current	Precipitation of Wettest Quarter	-0.175	0.129	0.255	-0.048	0.082
Current	Precipitation of Driest Quarter	-0.106	0.253	-0.189	-0.120	-0.206
Current	Precipitation of Warmest Quarter	-0.123	0.178	0.218	-0.169	-0.091
Current	Precipitation of Coldest Quarter	-0.131	0.178	-0.071	-0.010	0.004
Future	Annual Mean Temperature	-0.203	-0.121	-0.068	-0.019	0.060
Future	Mean Diurnal Range	-0.037	-0.252	-0.015	-0.134	-0.491
Future	Isothermality	-0.201	-0.033	0.019	0.123	-0.181
Future	Temperature Seasonality	0.197	0.012	0.007	-0.296	0.069
Future	Max Temperature of Warmest Month	-0.149	-0.195	-0.111	-0.270	0.097
Future	Min Temperature of Coldest Month	-0.211	-0.061	-0.069	0.106	0.069
Future	Temperature Annual Range	0.190	-0.049	0.019	-0.330	-0.028
Future	Mean Temperature of Wettest Quarter	-0.156	-0.149	0.061	-0.253	0.103
Future	Mean Temperature of Driest Quarter	-0.197	-0.091	-0.142	0.080	0.038
Future	Mean Temperature of Warmest Quarter	-0.167	-0.169	-0.105	-0.232	0.151
Future	Mean Temperature of Coldest Quarter	-0.209	-0.086	-0.054	0.091	0.025
Future	Annual Precipitation	-0.165	0.203	0.118	-0.083	0.002
Future	Precipitation of Wettest Month	-0.166	0.130	0.289	-0.054	0.097
Future	Precipitation of Driest Month	-0.082	0.258	-0.203	-0.138	-0.232
Future	Precipitation Seasonality	-0.022	-0.209	0.382	0.071	-0.204
Future	Precipitation of Wettest Quarter	-0.168	0.138	0.277	-0.056	0.086
Future	Precipitation of Driest Quarter	-0.091	0.260	-0.192	-0.134	-0.217
Future	Precipitation of Warmest Quarter	-0.096	0.179	0.252	-0.191	-0.099
Future	Precipitation of Coldest Quarter	-0.137	0.177	-0.051	-0.017	0.022

References

1. R Core Team. R: A Language and Environment for Statistical Computing. (2019). Available at: https://www.r-project.org/.
2. Dutilleul, P., Clifford, P., Richardson, S. & Hemon, D. Modifying the t Test for Assessing the Correlation Between Two Spatial Processes. *Biometrics* **49**, 305 (1993).
3. Olson, D. M. *et al.* Terrestrial Ecoregions of the World: A New Map of Life on Earth. *Bioscience* **51**, 933–938 (2001).
