# Supplementary material for: Predicted climate shifts within terrestrial protected areas worldwide
Source: Nat Commun. 2019 Oct 21;10:4787. doi: 10.1038/s41467-019-12603-w (PMC6803628; doi:10.1038/s41467-019-12603-w)
Supplement: Supplementary file 3 — Reporting Summary [file 41467_2019_12603_MOESM3_ESM.pdf]

nature research

Corresponding author(s): DBPR NCOMMS-19-11069A

Last updated by author(s): Sep 13, 2019

Reporting Summary

Nature Research wishes to improve the reproducibility of the work that we publish. This form provides structure for consistency and transparency in reporting. For further information on Nature Research policies, see Authors & Referees and the Editorial Policy Checklist .

Statistics

For all statistical analyses, confirm that the following items are present in the figure legend, table legend, main text, or Methods section.

n/a	Confirmed	
☐	☒	The exact sample size (*n*) for each experimental group/condition, given as a discrete number and unit of measurement
☐	☒	A statement on whether measurements were taken from distinct samples or whether the same sample was measured repeatedly
☐	☒	The statistical test(s) used AND whether they are one- or two-sided<br>*Only common tests should be described solely by name; describe more complex techniques in the Methods section.*
☐	☒	A description of all covariates tested
☐	☒	A description of any assumptions or corrections, such as tests of normality and adjustment for multiple comparisons
☐	☒	A full description of the statistical parameters including central tendency (e.g. means) or other basic estimates (e.g. regression coefficient) AND variation (e.g. standard deviation) or associated estimates of uncertainty (e.g. confidence intervals)
☐	☒	For null hypothesis testing, the test statistic (e.g. *F*, *t*, *r*) with confidence intervals, effect sizes, degrees of freedom and *P* value noted<br>*Give P values as exact values whenever suitable.*
☒	☐	For Bayesian analysis, information on the choice of priors and Markov chain Monte Carlo settings
☒	☐	For hierarchical and complex designs, identification of the appropriate level for tests and full reporting of outcomes
☐	☒	Estimates of effect sizes (e.g. Cohen's *d*, Pearson's *r*), indicating how they were calculated

Our web collection on statistics for biologists contains articles on many of the points above.

Software and code

Policy information about availability of computer code

Data collection	We used the R software (version 3.3.3) to process the data.<br>R Core Team (2017). R: A language and environment for statistical computing. R Foundation for Statistical Computing, Vienna, Austria. URL https://www.R-project.org/.
Data analysis	We used the R code provided by Carroll et al. (2015) to calculate climate change.<br>Carroll, C., Lawler, J. J., Roberts, D. R., & Hamann, A. (2015). Biotic and Climatic Velocity Identify Contrasting Areas of Vulnerability to Climate Change. PLOS ONE, 10(10), e0140486. https://doi.org/10.1371/journal.pone.0140486

For manuscripts utilizing custom algorithms or software that are central to the research but not yet described in published literature, software must be made available to editors/reviewers. We strongly encourage code deposition in a community repository (e.g. GitHub). See the Nature Research guidelines for submitting code & software for further information.

Data

Policy information about availability of data

All manuscripts must include a data availability statement. This statement should provide the following information, where applicable:

- Accession codes, unique identifiers, or web links for publicly available datasets
- A list of figures that have associated raw data
- A description of any restrictions on data availability

All data used in this study is open. Data sources and data produced in this study are given in the main text and the supplementary information. See Carroll et al. [17] for R code.

Field-specific reporting

Please select the one below that is the best fit for your research. If you are not sure, read the appropriate sections before making your selection.

☐ Life sciences ☐ Behavioural & social sciences ☒ Ecological, evolutionary & environmental sciences

For a reference copy of the document with all sections, see nature.com/documents/nr-reporting-summary-flat.pdf

Ecological, evolutionary & environmental sciences study design

All studies must disclose on these points even when the disclosure is negative.

Study description	We calculated the proportion of novel and disappearing climate conditions inside each of 137,432 terrestrial protected areas by the year 2070.
Research sample	This study includes 137,432 terrestrial protected areas worldwide. The protected area data were provided by the World Database on Protected Area (WDPA).
Sampling strategy	We rasterized the protected area polygons in the same resolution as the climate data (30 arc seconds, i.e. approx. 900 m at the equator) via cell center coverage. Thus, relatively small PAs and PAs which have an elongated shape may cover only a few or even no raster cells. After rasterization, 137,735 PAs remained, from which another 303 PAs were excluded because the centroids of those 303 PAs were located in the 'Mangroves' biome and are consequently assumed to be coastal PAs.
Data collection	Protected area data are given by the World Database on Protected Area (WDPA, version January 2018, www.protectedplanet.org). Climate data are provided by the WorldClim project (Hijmans et al. 2005, www.worldclim.org). Biome and ecoregion data are provided by Olson et al. (2001); the elevation and terrain ruggedness index by Amatulli et al. (2018); the human footprint index by Venter et al. (2016); the irreplaceability index by Le Saout et al. (2013).
Timing and spatial scale	Protected area data are given by the World Database on Protected Area (WDPA), version January 2018. Current climate data represents the mean of the period 1960-1990. Future climate data represents the mean of the period 2061-2080. The human footprint index was calculated for the year 2009; the irreplaceability index for October 2012.
Data exclusions	We excluded coastal protected areas because we were not interested in marine environments.
Reproducibility	NA
Randomization	We assigned each PA to its biome by overlaying the PA centroids and the biome polygons provided by Olson et al. (2001).
Blinding	NA

Did the study involve field work? ☐ Yes ☒ No

Reporting for specific materials, systems and methods

We require information from authors about some types of materials, experimental systems and methods used in many studies. Here, indicate whether each material, system or method listed is relevant to your study. If you are not sure if a list item applies to your research, read the appropriate section before selecting a response.

Materials & experimental systems

n/a	Involved in the study
☒	☐ Antibodies
☒	☐ Eukaryotic cell lines
☒	☐ Palaeontology
☒	☐ Animals and other organisms
☒	☐ Human research participants
☒	☐ Clinical data

Methods

n/a	Involved in the study
☒	☐ ChIP-seq
☒	☐ Flow cytometry
☒	☐ MRI-based neuroimaging
