# Supplementary material for: Predicted climate shifts within terrestrial protected areas worldwide
Source: Nat Commun. 2019 Oct 21;10:4787. doi: 10.1038/s41467-019-12603-w (PMC6803628; doi:10.1038/s41467-019-12603-w)
Supplement: Supplementary file 4 — Description of Additional Supplementary Files [file 41467_2019_12603_MOESM4_ESM.pdf]

Description of Additional Supplementary Files

File Name: Supplementary Data 1

Description: Supplementary Data 1 lists all the PAs with corresponding WDPA IDs, climate change indices and PA characteristics as applied in this study. The coordinates x and y included in the spreadsheet are given in degree (latitude and longitude) while WGS84 was used as a coordinate reference system. The area is given in km^2, elevation and terrain ruggedness in m. Other characteristics do not hold any unit.
