# Supplementary material for: Predicted climate shifts within terrestrial protected areas worldwide
Source: Nat Commun. 2019 Oct 21;10:4787. doi: 10.1038/s41467-019-12603-w (PMC6803628; doi:10.1038/s41467-019-12603-w)
Supplement: Supplementary file 2 — Peer Review File [file 41467_2019_12603_MOESM2_ESM.pdf]

Reviewers' Comments:

Reviewer #1:
Remarks to the Author:
This study represents the application of previously-developed methods in a global context and to a new question, the degree of prevalence of novel climates in the global protected areas network. The question is of broad relevance, and the computational challenge to complete such an analysis is substantial, so I believe that the study has substantial merit.
The methods are generally sound, but I have several comments and suggested clarifications.
Line (ln) 75 and elsewhere: the species richness metric is referred to here as biotic uniqueness/irreplaceability based on the term defined in Le Saout et al. 2013. The term irreplaceability however is more commonly used for a different metric in the context of systematic conservation planning, and I didn't realize that here the term refers to a type of my rare species richness until it was defined ln 269-270. Please add a short (partial sentence) defining uniqueness when it is first mentioned near ln 75.
More importantly, the negative correlation between irreplaceability and disappearing/novel climates is likely driven by the opposing latitudinal gradients in anthropogenic warming and species richness (i.e. polar regions are warming faster and are less speciose). This should be noted in the discussion.
ln 84: decreasing environmental heterogeneity only results in increased magnitude of climate change at the local (i.e. microrefugial) scale, so the analogy in this sentence needs to be explained better.
Discussion: One or more additional sentences are needed at the very beginning of the discussion to contextualize the results and/or review why the study's questions are important.
I think all of themes highlighted in the discussion are valid points, however I think the authors could have expanded this section somewhat by placing the results in context of previous work.
ln 147: orobiome should be defined
Methods: The bin width used by Carroll et al. (2 PCA units) is contingent on the number of PCA axes analyzed as well as other factors. For example, later studies analyzing climate types defined over only 2 PCA axes (Carroll et al. 2017, reference 12 in this study) used a bin width of only 0.25 PCA units. A sensitivity analysis is usually required, as was done here (Fig S5), so this is not a shortcoming of the current study, rather a suggestion to give a better explanation in the methods.
The manuscript needs extensive stylistic editing and rephrasing in many places, e.g. ln 83 "risk to lose uniqueness" should be "risk of losing uniqueness". But a complete stylistic edit is beyond the scope of this peer review and will require work by an editor or author.

Reviewer #2:
Remarks to the Author:
This study describes a very "large n" (~130,000) analysis of climate change impacts on nearly all of the worlds protected areas (PAs), for which the authors deserve considerable accolades. In terms of novelty, several other studies have tackled similar analyses at a global scale, though for biodiversity hotspots (Bellard et al. (2014) GEB) or ecoregions (Beaumont et al. (2011) PNAS). Neither of these studies is cited, BTW. Overall, I think this paper is of broad interest, but it suffers from a few issues that require attention. I place these in two board categories: (1) the methods used to calculate climate change impacts and (2) writing / presentation.

Starting with issues related to Methods…

I find the methods used to estimate climate novelty / disappearing climates to be highly convoluted. Perhaps this is because of lack of clear explanation rather than an issue with the methods themselves

per se. The authors do not explain why binning is used in the first place rather than a traditional 'distance-based' method of calculating novelty as nearly all other studies have done except those cited by the authors. After going to the trouble of reading the cited publications, (Carroll et al, and Hammann et al.), it appears that binning is used to overcome the substantial computational burdens of calculating pairwise distances between so many grid cells. It would be nice for the authors to explain this better & state why they are not using established distance-based methods.

While I appreciate the computational issues, I have a few issues with the binning method. First, as far as I can tell, it does not produce any measure of climate dissimilarity. Rather, simply indicates a yes/no change in existing climate class – which means a cell may change to a distant class, but it is weighted the same as a cell that changes to a new. But adjacent class – correct? Second, *and this is crucial*, there is no scaling of climate dissimilarity relative to historic interannual variability. This means that climate change within a PA with highly variable historic climate will have the same weighting as a PA with very stable historic climate.

Finally, it is completely unclear to me which pools of climate are being compared. I believe it is climate within PAs only – it would be nice if this was clearly stated up front. To do this analysis correctly, one should compare the climate within each PA to (1) all climates globally (hard / unreasonable computationally and perhaps not relevant from an ecological / conservation point of view) or better yet (2) climates within a reasonable distance of each PA and (3) climates within all PAs. This would provide a more comprehensive estimate of how climate change will impact PAs, where climate change will render climate within PAs novel / disappearing globally, regional, or only relative to the existing PA network. It would be possible to incorporate global land cover to ensure that only "unmodified" grid cells are used in the climate pool. A distance based method could be used if coarser grain climate data are used for areas outside PAs, which also would eliminate the need for the onerous and problematic binning method.

L296: What percent of the total current & future cells is 10,000,000? Did you do this subsampling once or multiple times. How representative is this sample of the entire climate space? Did the sample account for the fact that certain climate are over / under represented?

L315-322: Is it appropriate to use bins of constant width or should bin width be set as a function quantiles?

L324-328: I couldn't follow much of this.

L345,346: "overrate" * "underrate"? Should these be overestimate / underestimate?

Moving on to potential issues with the writing / presentation…

I felt the paper read a bit as if it were hastily prepared with little attention to detail, clarity, or grammar. It also often lacked important details (e.g., critical aspects of the methods often were not clearly explained or the reader was expected to read other publications to obtain the necessary information). Taken together, oversights, grammatical errors, and lack of clear writing made the paper hard / frustrating to follow at times.

L156-158 is a good example of a sentence that is difficult to unpack: "Besides the spatially differentiated degree of climate change between biomes, we found the numbers and sizes of PAs within biomes to explain their projected magnitude of disappearing and novel climate." Another is L202-204: "Moreover, the components of biodiversity respond individually to various climate change

metrics, but modifying range size and location are the major types of response." I'm not really sure what either of these sentences mean. In addition to addressing a number of grammatical issues, given the wide readership of Nature Communications, I think the writing needs to be improved – especially to add clear, descriptive sentences of the methods.

L19: Most reader's will not know that 'business-as-usual' means continued high emission / high warming.

L32-33: not clear what is meant by "species dislocate" = 'shift their range'?

L40-41: Why isn't the resolution of climate change maps also a problem for the present study?

L45-50: This text is key as it constitutes the only explanation of the methods in the main paper. However, it is highly confusing – mainly because it is not clear exactly which climate domains are being prepared. What does "relative to the individual climate pool of each PA" actually mean? From reading the methods, my understand is these metrics are calculated from each PA relative to the *entire pool* of climates across all PAs (but not the entire globe) – correct? The novelty / disappearing descriptions are also unclear. Again, which 'climate pools' are being compared for each metric? In this section – or a separate paragraph – it needs to made clear that this study is not looking at the global climate domain (or even climates within a given radius of each PA), only climates within PAs.

L52: Brief description of the algorithm would be much appreciated here. It seems strange to include details about what an RCP is, but nothing about the methods used to calculate climate impacts.

L52: "Other than"? Should this be "Rather than"?

L57: The mean and standard deviation of what? Proportion of raster cells that are novel / disappearing?

L72: What would be considered a "high degree of climate change"?

L74: What is an "elevational level"? Is this elevation, range of elevation, etc?

L91: "By" 2070? This sentence is hard to follow. What does it mean for an individual PA to experience 40% of disappearing climates? Is this trying to say that on average, 40% of the climates in existing PAs will disappear by 2070?

L96-97: Figure of results for this statement?

FIGURE 2 – It appears that there are very few locations with an index near 1, so it is hard to see spatial variation as most of the map is blue or green. Also, what is the "disappearing climate index"? I don't think this is ever defined. Why not use proportion like in the text?

L114-116: Here is a good example of where an important result is left for the reader to sort through in terms of interpreting the results. Here the reader is expected to refer to the Methods & Supplementary Information to determine why the disappearing climate index may over- or underestimate ecological change associated with climate change. Why not just tell the reader why this may be the case instead of sending them to the supplements?

FIGURE 3 – Not clear what the y-axes show in this figure – is it the disappearing climate INDEX?

L146…: This seems like a strange intro line for the discussion which focusses on mountains and a very specific result for this biome rather than summarizing the overall findings and their importance.

Point-by-point response to the reviewers' comments on the manuscript NCOMMS-19-11069-T

Dear Reviewer #1

We are very grateful for your helpful comments. You provided in-depth recommendations on how to improve the manuscript, especially concerning the methods and writing. We appreciate the time and effort that you put into evaluating our manuscript.

We have responded (bold font) to each of your comments below and explain in detail the changes we have made to the manuscript. Our manuscript has improved overall, thanks to the constructive criticisms from this round of review.

Sincerely,

the Authors

Reviewer #1 (Remarks to the Author):

This study represents the application of previously-developed methods in a global context and to a new question, the degree of prevalence of novel climates in the global protected areas network. The question is of broad relevance, and the computational challenge to complete such an analysis is substantial, so I believe that the study has substantial merit.

The methods are generally sound, but I have several comments and suggested clarifications.

Line (ln) 75 and elsewhere: the species richness metric is referred to here as biotic uniqueness/irreplaceability based on the term defined in Le Saout et al. 2013. The term irreplaceability however is more commonly used for a different metric in the context of systematic conservation planning, and I didn't realize that here the term refers to a type of my rare species richness until it was defined ln 269-270. Please add a short (partial sentence) defining uniqueness when it is first mentioned near ln 75.

Our answer: Done (L107ff).

More importantly, the negative correlation between irreplaceability and disappearing/novel climates is likely driven by the opposing latitudinal gradients in anthropogenic warming and species richness (i.e. polar regions are warming faster and are less speciose). This should be noted in the discussion.

Our answer: Done (L261ff).

ln 84: decreasing environmental heterogeneity only results in increased magnitude of climate change at the local (i.e. microrefugial) scale, so the analogy in this sentence needs to be explained better.

Our answer: We have reformulated the sentence as follows (L101): "We expect the degree of climate change inside individual PAs to decrease with increasing environmental heterogeneity (i.e. with decreasing PA size, decreasing elevation and decreasing terrain ruggedness). This is because completely novel/disappearing climates are less likely under high environmental and climate diversity."

Discussion: One or more additional sentences are needed at the very beginning of the discussion to contextualize the results and/or review why the study's questions are important.

Our answer: Done. We added an entire paragraph to the Discussion section. See first paragraph of the Discussion section (L201ff).

I think all of themes highlighted in the discussion are valid points, however I think the authors could have expanded this section somewhat by placing the results in context of previous work.

Our answer: Done. We added an entire paragraph to the Discussion section highlighting nine previous studies on global climate change patterns. See third paragraph of the Discussion section (L217ff).

ln 147: orobiome should be defined

Our answer: Modified to 'mountain biomes' (L236).

Methods: The bin width used by Carroll et al. (2 PCA units) is contingent on the number of PCA axes analysed as well as other factors. For example, later studies analysing climate types defined over only 2 PCA axes (Carroll et al. 2017, reference 12 in this study) used a bin width of only 0.25 PCA units. A sensitivity analysis is usually required, as was done here (Fig S5), so this is not a shortcoming of the current study, rather a suggestion to give a better explanation in the methods.

Our answer: We rephrased the corresponding paragraph in the Methods to clarify our approach (L430ff).

The manuscript needs extensive stylistic editing and rephrasing in many places, e.g. ln 83 "risk to lose uniqueness" should be "risk of losing uniqueness". But a complete stylistic edit is beyond the scope of this peer review and will require work by an editor or author.

Our answer: We have stylistically edited the entire manuscript.

Dear Reviewer #2

We are very grateful for your helpful comments. You provided in-depth recommendations on how to improve the manuscript, especially concerning the methods and writing. We appreciate the time and effort that you put into evaluating our manuscript.

We have responded (bold font) to each of your comments below and explain in detail the changes we have made to the manuscript. Our manuscript has improved overall, thanks to the constructive criticisms from this round of review.

Sincerely,

the Authors

Reviewer #2 (Remarks to the Author):

This study describes a very "large n" (~130,000) analysis of climate change impacts on nearly all of the worlds protected areas (PAs), for which the authors deserve considerable accolades. In terms of novelty, several other studies have tackled similar analyses at a global scale, though for biodiversity hotspots (Bellard et al. (2014) GEB) or ecoregions (Beaumont et al. (2011) PNAS). Neither of these studies is cited, BTW. Overall, I think this paper is of broad interest, but it suffers from a few issues that require attention. I place these in two board categories: (1) the methods used to calculate climate change impacts and (2) writing / presentation.

Our answer: Thank you very much for the literature recommendations! Both publications are cited in the revised version as they are relevant for our work. We added an entire paragraph that relates our work to previous studies, as it was requested by Reviewer #1 as well (L217ff).

Starting with issues related to Methods…

I find the methods used to estimate climate novelty / disappearing climates to be highly convoluted. Perhaps this is because of lack of clear explanation rather than an issue with the methods themselves per se. The authors do not explain why binning is used in the first place rather than a traditional 'distance-based' method of calculating novelty as nearly all other studies have done except those cited by the authors. After going to the trouble of reading the cited publications, (Carroll et al, and Hamann et al.), it appears that binning is used to overcome the substantial computational burdens of calculating pairwise distances between so many grid cells. It would be nice for the authors to explain this better & state why they are not using established distance-based methods.

Our answer: We apologize for the unclear explanation. The binning method is a distance-based method but not a linear one such as the Euclidean distance. The standardized Euclidean distance was applied in the classic work of Williams et al. (Williams, Jackson, & Kutzbach, 2007) **that we did cite (L69). Moreover, the binning method is an established method (see** Hamann et al. 2015; Batllori et al. 2017; Carroll et al. 2017)**. Therefore, we suggest to keep the binning method as provided by Carroll et al.** (Carroll, Lawler, Roberts, & Hamann, 2015)**. The binning method implies pros and cons such as each other method. Some cons are highlighted by Reviewer #2. We are aware of these disadvantages and provide better explanations as Reviewer #2 suggested (L68-71,**

L285-300, L420-423). These drawbacks include a rather basic measurement of climate change but for the benefit of computational feasibility when high-resolution data are used. We have added a discussion about its advantages and disadvantages compared with other methods in the Methods section (L420-428).

While I appreciate the computational issues, I have a few issues with the binning method. First, as far as I can tell, it does not produce any measure of climate dissimilarity. Rather, simply indicates a yes/no change in existing climate class – which means a cell may change to a distant class, but it is weighted the same as a cell that changes to a new. But adjacent class – correct? Second, *and this is crucial*, there is no scaling of climate dissimilarity relative to historic interannual variability. This means that climate change within a PA with highly variable historic climate will have the same weighting as a PA with very stable historic climate.

Our answer: Reviewer #2 is right in both points. The binning method does not measure climate dissimilarity in an ordinal way. The binning method does not standardize the future climate anomaly regarding interannual climate variability. These are weaknesses of the binning approach, which we have to accept for the benefit of computational feasibility. Therefore, we have explained these drawbacks in the revised version (L420-428) and added a discussion about the meaning of interannual climate variability L292-300.

We clearly explain the importance of past interannual climate variability: Ecosystems that have experienced high, historical interannual climate variability are expected to be more resilient to climate change (L298). However, interannual climate variability is rarely incorporated into algorithms that search for climate analogs (Garcia, Cabeza, Rahbek, & Araujo, 2014). The reasons for this fact remain unknown but we suppose this is because the relevance of interannual climate variability is not yet fully understood (Holmgren, Hirota, Van Nes, & Scheffer, 2013), the quality (i.e. the temporal and spatial resolution) of data on interannual climate variability might be insufficient and the computational burdens are enormous.

Thus, we hope we could dispel the Reviewer's doubts about the binning approach. We decided to keep the binning approach because Reviewer #2 mentioned that he or she was not familiar with the binning approach and because Reviewer #2 did not demand a different methodological approach. However, we would be able to apply a different algorithm such as the standardized Euclidean distance incorporating interannual climate variability.

Finally, it is completely unclear to me which pools of climate are being compared. I believe it is climate within PAs only – it would be nice if this was clearly stated up front.

Our answer: This is clearly stated at the end of the introduction (L45ff) and in the methods (L384ff).

To do this analysis correctly, one should compare the climate within each PA to (1) all climates globally (hard / unreasonable computationally and perhaps not relevant from an ecological / conservation point of view) or better yet (2) climates within a reasonable distance of each PA and (3) climates within all PAs. This would provide a more comprehensive estimate of how climate change will impact PAs, where climate change will render climate within PAs novel / disappearing globally, regional, or only relative to the existing PA network. It would be possible to incorporate global land cover to ensure that only "unmodified" grid cells are used in the climate pool. A distance based method could be used if coarser grain climate data are used for areas outside PAs, which also would eliminate the need for the onerous and problematic binning method.

Our answer: In the new version, we have calculated novel and disappearing climate of each PA at a local, regional and global scale. For the local scale, the novel climate index was quantified by the proportion of raster cells of a single PA that hold climate classes in the future scenarios but are currently not present inside the same single PA (i.e. local-scale). For the regional scale, the novel climate index was quantified by the proportion of raster cells of a single PA that hold climate classes in the future scenarios but are currently not present inside the entire PA network of the biome (i.e. regional-scale). For the global scale, the novel climate index was quantified by the proportion of raster cells of a single PA that hold climate classes in the future scenarios but are currently not present inside the global PA network (i.e. global-scale). The disappearing climate index was calculated by using the proportion of raster cells inside a single PA that hold climate classes currently but are absent in the future scenarios. We considered only grid cells of protected area because of our limited computing capacity. Because the protected area network is more and more seen as the only remaining safe site for biodiversity conservation (Jackson & Gaston, 2008; Pimm, Jenkins, & Li, 2018)**, we find this approach reasonable from a conservation perspective.**

In other words, the reviewer's recommendation (1) was not feasible for us due to our limited computing capacity. Reviewer #2 even mentions that recommendation (1) might not be possible nor relevant. Recommendation (2) was feasible for us with few limitations: For the regional scale, we compared the change of climate classes within individual protected areas to the pool of climate classes of all protected areas within the biome. We used biomes instead of any subjective distance around protected area, because climates that are new to or disappear from biomes indicate significant ecological changes (Williams et al., 2007)**. We were able to conduct the Reviewer's third suggestion (3).**

Due to the additional analyses for the regional and global scale, we had to add and rearrange most parts of the manuscript (see new manuscript version). Tracking changes in the manuscript file would have been a mess. Now we focus on novel climate in the main text because the novel climate index showed more interesting patterns at the regional and global scale than the disappearing climate index. But note that the novel and disappearing index are highly correlated, suggesting very similar geographical patterns of the indices.

Moreover, it is unclear to us what you mean with "unmodified" cells. Modified land such as semi-natural landscapes are included in many PAs (e.g. UNESCO Man and Biosphere Reserves, Nature Parks, etc.). Such land can be very relevant for biodiversity conservation, particularly in regions with otherwise high human land use such as in Europe. The definition of "unmodified" is highly controversial. Hence, a reduction of the data to unmodified cells would unnecessarily complicate the analysis. We consequently decided against this selection.

We agree with Reviewer #2 that "a distance based method could be used if coarser grain climate data are used for areas outside PAs, which also would eliminate the need for the onerous and problematic binning method." However, this would mean we had to apply a totally different method. We are reluctant to mix two substantially different climate change algorithms in one single manuscript about such a large dataset. This would confuse the reader. Nevertheless, we see great potential for another manuscript using the other distance-based approach.

L296: What percent of the total current & future cells is 10,000,000? Did you do this subsampling once or multiple times. How representative is this sample of the entire climate space? Did the sample account for the fact that certain climate are over / under represented?

Our answer: The 10,000,000 cells represent 40% of the total number of cells (L396). The subsampling is done once per future climate model (20 models in total). Including more cells does drastically increase the computing time but only marginally increase the variation in the climate

data that can be explained by the PCA axes. This is why we decided on the representative subsample of 10,000,000 cells. The random sampling implies that each climate type is sampled proportionally to its extent. This approach is analog to Batllori et al. (Batllori et al., 2017)**.**

L315-322: Is it appropriate to use bins of constant width or should bin width be set as a function quantiles?

Our answer: We applied an established algorithm that uses constant bin widths. The bin widths as a function of quantiles would mean that the rare climate is located in smaller bins. Thus, rare climate cells will change bins first, given a constant climate shift across all climates. Consequently, the novel and disappearing climate index of PAs with rare climate would be enhanced. We think that such modifications would make the results much harder to interpret.

L324-328: I couldn't follow much of this.

Our answer: We have substantially rephrased this part (L430ff) and removed all the redundant information.

L345,346: "overrate" * "underrate"? Should these be overestimate / underestimate?

Our answer: Yes. Fixed (L457ff).

Moving on to potential issues with the writing / presentation…

I felt the paper read a bit as if it were hastily prepared with little attention to detail, clarity, or grammar. It also often lacked important details (e.g., critical aspects of the methods often were not clearly explained or the reader was expected to read other publications to obtain the necessary information). Taken together, oversights, grammatical errors, and lack of clear writing made the paper hard / frustrating to follow at times.

Our answer: We guarantee that the manuscript was not hastily written. We apologize for the lack of clarity, grammatical mistakes and missing detail. The abbreviation of a previous manuscript version has probably caused these shortcomings. We think all of these aspects have substantially improved in the new version.

L156-158 is a good example of a sentence that is difficult to unpack: "Besides the spatially differentiated degree of climate change between biomes, we found the numbers and sizes of PAs within biomes to explain their projected magnitude of disappearing and novel climate."

Our answer: Yes, this is a grammatical error. Fixed (L148ff).

Another is L202-204: "Moreover, the components of biodiversity respond individually to various climate change metrics, but modifying range size and location are the major types of response." I'm not really sure what either of these sentences mean.

Our answer: Fixed (L288ff). Now this part reads "Here we highlight only novel and disappearing climate conditions, but there are many more dimensions of climate change to which species react."

In addition to addressing a number of grammatical issues, given the wide readership of Nature Communications, I think the writing needs to be improved – especially to add clear, descriptive sentences of the methods.

Our answer: We have worked the writing substantially.

L19: Most reader's will not know that 'business-as-usual' means continued high emission / high warming.

Our answer: We have modified this sentence to provide this information (L84ff).

L32-33: not clear what is meant by “species dislocate” = ‘shift their range’?

Our answer: Yes, it is. We changed that to “shift” (L31).

L40-41: Why isn’t the resolution of climate change maps also a problem for the present study?

Our answer: We considered the global protected area and used climate data with a resolution of 1 km. Available maps on global climate change impact used data with a much coarser resolution but considered the global land area. Applying a resolution of 1 km to global land area would require huge computing capacity, which is not given. Hence, one has to either reduce the area or the resolution. We used the highest resolution available and focused on the global PA area.

L45-50: This text is key as it constitutes the only explanation of the methods in the main paper. However, it is highly confusing – mainly because it is not clear exactly which climate domains are being prepared. What does “relative to the individual climate pool of each PA” actually mean? From reading the methods, my understand is these metrics are calculated from each PA relative to the *entire pool* of climates across all PAs (but not the entire globe) – correct? The novelty / disappearing descriptions are also unclear. Again, which ‘climate pools’ are being compared for each metric? In this section – or a separate paragraph – it needs to made clear that this study is not looking at the global climate domain (or even climates within a given radius of each PA), only climates within PAs.

Our answer: This evaluation is very helpful. Thank you very much! We reformulated the text accordingly (L42-76).

L52: Brief description of the algorithm would be much appreciated here. It seems strange to include details about what an RCP is, but nothing about the methods used to calculate climate impacts.

Our answer: Done (L67-76).

L52: “Other than”? Should this be “Rather than”?

Our answer: This part reads now: “In contrast to other linear, distance-based climate change algorithms (e.g. Williams et al. 14), this approach …” (L68ff).

L57: The mean and standard deviation of what? Proportion of raster cells that are novel / disappearing?

Our answer: Fixed (L80ff): “We show the mean and standard deviation of the PAs’ novel and disappearing climate index throughout the ten Global Climate Models (GCM) for each RCP scenario.”

L72: What would be considered a “high degree of climate change”?

Our answer: This expression was confusing. We removed this expression (L95ff).

L74: What is an “elevational level”? Is this elevation, range of elevation, etc?

Our answer: We calculated the median elevation of each PA (see Methods L343). We write “elevation” in the new version.

L91: “By” 2070? This sentence is hard to follow. What does it mean for an individual PA to experience 40% of disappearing climates? Is this trying to say that on average, 40% of the climates in existing PAs will disappear by 2070?

Our answer: We have rephrased the sentence (L118ff). On global average, 40% of the climate inside any individual PA will disappear until 2070. Please note that we focus on novel climate in the new manuscript version because the patterns of the regionally and globally novel climates were more interesting than the disappearing climate patterns.

L96-97: Figure of results for this statement?

Our answer: L121ff: There is no need for a figure because these numbers are results of correlations between the two indices. The correlation results are given in brackets.

FIGURE 2 – It appears that there are very few locations with an index near 1, so it is hard to see spatial variation as most of the map is blue or green. Also, what is the "disappearing climate index"? I don't think this is ever defined. Why not use proportion like in the text?

Our answer: We have tried many different point sizes to mark the PAs on the global map. When we further reduce the point size, the colours are hard to see. When we increase the point size, the points largely overlap and are hard to separate. The main pattern is most pronounced with the current point size.

The disappearing climate index is defined at the end of the Introduction (L63ff) and in the Methods (L482ff).

L114-116: Here is a good example of where an important result is left for the reader to sort through in terms of interpreting the results. Here the reader is expected to refer to the Methods & Supplementary Information to determine why the disappearing climate index may over- or underestimate ecological change associated with climate change. Why not just tell the reader why this may be the case instead of sending them to the supplements?

Our answer: We agree on this point. We have listed the biomes (L141ff).

FIGURE 3 – Not clear what the y-axes show in this figure – is it the disappearing climate INDEX?

Our answer: Yes. This is also mentioned in the figure caption.

L146…: This seems like a strange intro line for the discussion which focusses on mountains and a very specific result for this biome rather than summarizing the overall findings and their importance.

Our answer: You are right. We have changed the beginning of the discussion considerably (L201ff).

References

Batllori, E., Parisien, M.-A., Parks, S. A., Moritz, M. A., & Miller, C. (2017). Potential relocation of climatic environments suggests high rates of climate displacement within the North American protection network. *Global Change Biology*, *23*(8), 3219–3230. https://doi.org/10.1111/gcb.13663

Carroll, C., Lawler, J. J., Roberts, D. R., & Hamann, A. (2015). Biotic and Climatic Velocity Identify Contrasting Areas of Vulnerability to Climate Change. *PLOS ONE*, *10*(10), e0140486. https://doi.org/10.1371/journal.pone.0140486

Carroll, C., Roberts, D. R., Michalak, J. L., Lawler, J. J., Nielsen, S. E., Stralberg, D., … Wang, T. (2017). Scale-dependent complementarity of climatic velocity and environmental diversity for identifying priority areas for conservation under climate change. *Global Change Biology*, *23*(11), 4508–4520. https://doi.org/10.1111/gcb.13679

Hamann, A., Roberts, D. R., Barber, Q. E., Carroll, C., & Nielsen, S. E. (2015). Velocity of climate change algorithms for guiding conservation and management. *Global Change Biology*, *21*(2), 997–1004. https://doi.org/10.1111/gcb.12736

Jackson, S. F., & Gaston, K. J. (2008). Land use change and the dependence of national priority species on protected areas. *Global Change Biology*. https://doi.org/10.1111/j.1365-2486.2008.01628.x

Pimm, S. L., Jenkins, C. N., & Li, B. V. (2018). How to protect half of earth to ensure it protects sufficient biodiversity. *Science Advances*, *4*(8), 1–9. https://doi.org/10.1126/sciadv.aat2616

Williams, J. W., Jackson, S. T., & Kutzbach, J. E. (2007). Projected distributions of novel and disappearing climates by 2100 AD. *Proceedings of the National Academy of Sciences of the United States of America*, *104*(14), 5738–5742. https://doi.org/10.1073/pnas.0606292104

Reviewers' Comments:

Reviewer #1:
Remarks to the Author:
Generally speaking, the authors have addressed the issues identified by reviewers.
I believe that there is an error in lines 101-104:
“We expect the degree of climate change inside individual PAs to decrease with increasing environmental heterogeneity (i.e. with decreasing PA size, decreasing elevation and decreasing terrain ruggedness).”
I believe that the correct text would be:
“We expect the degree of climate change inside individual PAs to decrease with increasing environmental heterogeneity (i.e. with increasing PA size, increasing elevation and increasing terrain ruggedness).” OR
“We expect the degree of climate change inside individual PAs to increase with decreasing environmental heterogeneity (i.e. with decreasing PA size, decreasing elevation and decreasing terrain ruggedness).”
Also, minor point: the text lines 404-405: “These first five axes mainly correspond to thermal variables” seems an inaccurate summary of the PCA, as some axes eg axis 2 have substantial contributions from precipitation-related variables.
Also, although the manuscript is improved in this since the first version, it still contains many stylistic (ie grammatical) errors. If the journal editorial process does not provide stylistic editing, and none of the coauthors can provide this service, I would suggest that the authors consider paying for assistance from such a service. As it stands, this lack of grammatical editing will present a barrier to some readers.

Reviewer #2:
Remarks to the Author:
I was R2 of the original manuscript. I feel the authors have done a thorough job addressing my concerns as well as those raised by R1. Other than addressing a few minor grammatical / wording issues = throughout the MS, I do not have any additional suggested changes.

Point-by-point response to the reviewers' comments on the manuscript NCOMMS-19-11069A

Dear Reviewer #1

We are very thankful for your in-depth review of our manuscript. We appreciate the time and effort that you put into improving our manuscript. We have responded (bold font) to each of your comments below and explain in detail the changes we have made to the manuscript in the second round of review.

Sincerely,

the Authors

Reviewer #1 (Remarks to the Author):

Generally speaking, the authors have addressed the issues identified by reviewers.

I believe that there is an error in lines 101-104:

"We expect the degree of climate change inside individual PAs to decrease with increasing environmental heterogeneity (i.e. with decreasing PA size, decreasing elevation and decreasing terrain ruggedness)."

I believe that the correct text would be:

"We expect the degree of climate change inside individual PAs to decrease with increasing environmental heterogeneity (i.e. with increasing PA size, increasing elevation and increasing terrain ruggedness)." OR

"We expect the degree of climate change inside individual PAs to increase with decreasing environmental heterogeneity (i.e. with decreasing PA size, decreasing elevation and decreasing terrain ruggedness)."

Our answer: Reviewer #2 is right. We apologize for that mistake and changed that sentence accordingly (L105).

Also, minor point: the text lines 404-405: "These first five axes mainly correspond to thermal variables" seems an inaccurate summary of the PCA, as some axes eg axis 2 have substantial contributions from precipitation-related variables.

Our answer: We agree. This sentence now reads "These first five axes correspond to thermal and hydraulic variables (Supplementary Table S1 and S2)."

Also, although the manuscript is improved in this since the first version, it still contains many stylistic (ie grammatical) errors. If the journal editorial process does not provide stylistic editing, and none of the coauthors can provide this service, I would suggest that the authors consider paying for assistance from such a service. As it stands, this lack of grammatical editing will present a barrier to some readers.

Our answer: We apologize for any stylistic error and are very grateful for the stylistic editing by the Senior Editor Dr. Alexa McKay.

Dear Reviewer #2

We appreciate the time and effort that you put into evaluating our manuscript. Our manuscript has significantly improved, thanks to the constructive criticisms from your review.

Sincerely,

the Authors

Reviewer #2 (Remarks to the Author):

I was R2 of the original manuscript. I feel the authors have done a thorough job addressing my concerns as well as those raised by R1. Other than addressing a few minor grammatical / wording issues = throughout the MS, I do not have any additional suggested changes.

Our answer: We apologize for any stylistic error and are very grateful for the stylistic editing by the Senior Editor Dr. Alexa McKay.
